# Porifera Lectins: Diversity, Physiological Roles and Biotechnological Potential

**DOI:** 10.3390/md13085059

**Published:** 2015-08-07

**Authors:** Johan Gardères, Marie-Lise Bourguet-Kondracki, Bojan Hamer, Renato Batel, Heinz C. Schröder, Werner E. G. Müller

**Affiliations:** 1 Unité Molécules de Communication et Adaptation des Microorganismes, UMR 7245 CNRS, Muséum National d’Histoire Naturelle, CP 54, 57 rue Cuvier, Paris 75005, France; E-Mails: jgarderes@mnhn.fr (J.G.); bourguet@mnhn.fr (M.-L.B.-K.); 2 Laboratory for Marine Molecular Biology, Center for Marine Research, Ruđer Bošković Institute, G. Paliaga 5, 52210 Rovinj, Croatia; E-Mails: hamer@cim.irb.hr (B.H.); renato.batel@irb.hr (R.B.); 3 ERC Advanced Investigator Grant Research Group at Institute for Physiological Chemistry, University Medical Center of Johannes Gutenberg University Mainz, Duesbergweg 6, Mainz D-55128, Germany; E-Mail: hschroed@uni-mainz.de

**Keywords:** porifera, lectin, physiological roles, bioactivities

## Abstract

An overview on the diversity of 39 lectins from the phylum Porifera is presented, including 38 lectins, which were identified from the class of demosponges, and one lectin from the class of hexactinellida. Their purification from crude extracts was mainly performed by using affinity chromatography and gel filtration techniques. Other protocols were also developed in order to collect and study sponge lectins, including screening of sponge genomes and expression in heterologous bacterial systems. The characterization of the lectins was performed by Edman degradation or mass spectrometry. Regarding their physiological roles, sponge lectins showed to be involved in morphogenesis and cell interaction, biomineralization and spiculogenesis, as well as host defense mechanisms and potentially in the association between the sponge and its microorganisms. In addition, these lectins exhibited a broad range of bioactivities, including modulation of inflammatory response, antimicrobial and cytotoxic activities, as well as anticancer and neuromodulatory activity. In view of their potential pharmacological applications, sponge lectins constitute promising molecules of biotechnological interest.

## 1. Introduction

Lectins are widespread in nature and have been reported from prokaryotes, as well as from eukaryotes. They are defined as proteins or glycoproteins that recognize carbohydrates. The term of lectin appeared at the end of the nineteenth century as molecules able to recognize the histoblood group of antigens. The name lectin, related to the Latin verb “legere”, which means “to select”, was given by Sharpey and Boyd for describing a protein able to link non-covalently and reversibly to carbohydrates and agglutinate and/or precipitate polysaccharides and glycoproteins [1]. This definition has continuously evolved. Nowadays, a lectin is a protein or a glycoprotein, which differs from an immunoglobulin, with at least one carbohydrate recognition domain. The binding of the protein to carbohydrates does not involve any enzymatic modification or any cleavage of these carbohydrates. The interest of lectins resulted in their biological activities, their involvement in tissue morphogenesis, regulation of physiological functions and metabolism or protection against the environment [2,3,4,5,6]. Efforts in lectin purification were mainly performed in order to explore their biological activities and/or biotechnological potencies.

Plant lectins were intensively studied, especially the *Canavalia* spp. Concanavalin A and the *Phaseolus* spp. phytohaemagglutinins, because of their relevant proinflammatory and/or anticancer activities, as well as mitogenic effects on lymphocytes [7,8]. Plant lectins, in particular Concanavalin A, demonstrated to affect both apoptosis and autophagy by modulating cell-signaling pathways of cancer cell lines. Concanavalin A showed to collapse the potential of mitochondrial membrane in human melanoma A375 cells, triggering the release of the cytochrome C and the caspase activation, both involved in cell apoptosis. Concanavalin A also induced apoptosis in ovarian cancer SKOV3 cells by modulating the expression of apoptosis-involved proteins cyclooxygenase 2 (COX-2), B-cell lymphoma 2 (Bcl-2), and serine-threonine protein kinase AKT and activating the Foxola-Bim signaling pathways [9]. The bean *Phaseolus vulgaris* lectin demonstrated to inhibit the proliferation of nasopharyngeal carcinoma HONE-1 cells [10]. Furthermore, plant lectins were also used as polyclonal activators of T-cells in order to study the *in vitro* lymphocyte functions. Concanavalin A and phytohaemagglutins revealed to bind membrane carbohydrates expressed on T-lymphocytes, and then to activate mitogenic response of lymphocytes and the production of cytokines [11,12].

Marine organisms have also produced lectins with diverse bioactivities, including proinflammatory modulation, as well as antiviral or antimicrobial activities [13].

The algae *Ticocarpus crinitus* and the mussel *Mytilus trossulus* lectins showed to enhance the synthesis of cytokines. The *T. crinitus* lectin exhibited an increase in dose dependent manner of tumor necrosis factor α (TNFα), interleukine 6 and interferon γ (INFγ) in human whole-blood cells [14]. The *M. trossulus* lectin, at a concentration of 5 and 80 μg/mL, showed a stimulating effect on spontaneous and lipopolysaccharides (LPS)-induced production of TNFα by human peripheral blood cells. However, a stimulating activity on spontaneous and induced production of IFNγ was only observed at a concentration of 80 μg/mL. The growth of lymphocytes was also stimulated by the mussel *M. trossulus* lectin [15].

Antiviral effects were also found in some red algae, ascidians and sea worm lectins. Griffithsin, isolated from the red algae *Griffithsia* spp., showed to reduce the entry of coronaviruses, as well as the hepatitis C and Japanese encephalitis viruses, by binding the carbohydrates of the viral envelop [16,17,18]. The *Kappaphycus alvarezii* and *Booglea coacta* red algae lectins showed to inhibit the profileration of the human immunodeficiency virus-I by using the same mechanism [19,20]. The ascidian *Didemnum ternatanum*, as well as the *Chaetopterus variopedatus* and the *Serpula vermicularis* sea worm lectins also demonstrated activities against human inmmunodeficiency virus-1 (HIV-1) [21]. Both *C. variopedatus* and *S. vermicularis* lectins showed to inhibit the production of viral p24 antigen and the cytopathic effect induced by HIV-1 [22,23].

The mussel *Crenomytilus grayanus* lectin, which is potentially involved in recognition and clearance of bacterial pathogens in the shellfish, showed to interact with Gram-positive and Gram negative bacteria. This activity, which was inhibited by carbohydrates, revealed the involvement of a lectin [24].

Therefore, as illustrated with these few examples, marine lectins represent medical valuable tools for medical and/or biotechnological applications, mostly inspired from their physiological roles within the organisms. Sponge lectins have been also intensively studied and have revealed promising pharmacological and biotechnological potentials.

The Porifera phylum is composed of more than 8500 described species, showing a widespread geographical distribution [25]. Thanks to their ability to synthesize a great variety of molecules with diverse roles in defense, communication or adaptation to the environment, sponges appeared as a source of molecules with potential biomedical applications [26,27]. The most relevant examples are illustrated with the marketed antiviral 9-β-d-arabinofuranosyladenine (ara-A or Vidarabine) [28] and the antileukemic arabinofuranosyl (ara C or cytarabine) [29], both inspired from natural nucleosides isolated from the Caribbean sponge *Cryptotethya crypta* (de Laubenfels, 1949) [30], as well as the natural antitumor eribulin mesylate from marine sponge *Halichondria okadai* (Fleming, 1828) [31,32]. More recently, an increasing interest in sponges was developed because these marine organisms also represent a model of a well-established microecosystem with a complex diversity of microorganisms, which proved to be stable and permanent.

The studies on sponge lectins really started in the 1980s with their capacities to agglutinate erythrocytes. Since these works, a wide range of bioactivities has been demonstated, including mitogenic, antimicrobial, cytotoxic and neuromodulatory activities. Their biological roles have been described in the involvement of defense mechanisms in order to protect sponges from their predators [33], in the recognition and removal of aged or altered glycoconjugates [34] or in the aggregation of sponge cells [35].

Recently, a review covered the properties and biological activities of 17 marine sponge lectins [36]. We now provide a full coverage of the 39 lectins isolated from sponges, ranging from their purification, biochemical properties to their physiological roles and biotechnological potential.

## 2. Classification

Lectins are usually classified according to their primary structure and their binding selectivity. Sponge lectins include galectins, C-type, tachylectin-like and F-type lectins (Table 1).

Galectins (or S-type lectins) are restricted to the animal kingdom and display a preferential binding-selectivity for galactoside residues. They were divided into 3 subfamilies on the basis of their structural architecture: the proto-, chimera- and tandem-repeat-type galectins [37]. The galectin prototypes are non-covalent dimers, each possessing two identical carbohydrate recognition domains. The chimera-type galectins have two distinct domains, an *N*-terminal collagen-like domain and a *C*-terminal galectin, which potentially binds non-sugar moieties to sugar moieties. Finally, two carbohydrate recognition domains linked by a functional linker peptide are specific for the tandem-repeat-type galectin. Nonetheless, Porifera galectins differ from the usual animal galectins because they harbor specific structural features. They can create large complex molecules in the presence of Ca^2+^ (not involved in the carbohydrate binding) and showed an extremely high affinity for *N*-acetyl-galactosamine-containing saccharides [38].

C-type lectins are widespread in bacteria, plants and animals. This class of lectins contains collectins (mannose-binding lectins (MBL), ficolin, conglutinin and pulmonary surfactant), proteoglycan core proteins, selectins, endocytic receptors and macrophage receptors. The binding of these proteins to their specific carbohydrate residues is conditioned by the presence of Ca^2+^ in the environment [39].

Tachylectin-like lectins are proteins, which display high similarities with the tachylectin 1/limulus L6 lectin [40]. They usually exhibited antimicrobial activity against prokaryotes by linking their membrane carbohydrates. They display six tachylectin tandem repeats [41,42].

F-type lectins constitute a recent group, characterized by one carbohydrate recognition domain and specific binding of fucose. Two subgroups can be distinguished according to the F-type domain, which can present either a single or a tandem-repeat carbohydrate recognition domain. In this family, Ca^2+^ does not play a crucial role in the binding activity but in stabilizing the protein [39].

The interest in sponge lectins arose with the study of sponge extracts, which demonstrated their ability to agglutinate erythrocytes [43,44,45]. Indeed, sponge extracts from 48 different species, collected in the Red Sea, in the Australian Barrier Reef and along the Floridian coasts, were investigated. Forty-two percent of these sponge extracts exhibited agglutinating properties to human erythrocytes of ABO group [46]. Similar percentages were reported from sponge extracts collected in the Adriatic Sea, confirming the ubiquitous character of lectins [47]. Thus far, 39 lectins were identified from the Porifera phylum (Table 1).

**Table 1:** Biochemical properties of sponge lectins.

Name	Species	Order (Class)	Size in kDa (Subunits)	Carbohydrates	pI	Cations	Disulfide Bridges	pH/T °C Activity
**Sponge galectins**								
CchG 1	*Cinachyrella sp.*	Spirophorida(D)	50.0 (4)	galactoside residues	nd	no	no	nd/<100 °C
CchG 2	*Cinachyrella sp.*	Spirophorida (D)	50.0 (4)	galactoside residues	nd	no	no	nd/<100 °C
GCG	*Geodia cydonium*	Astrophorida (D)	60.0 (4)	galactoside residues	4.4	Ca^2+^	no	nd/nd
HoL-30	*Halichondria okadai*	Halichondrida (D)	60.0 (2)	galactoside residues	6.7	no	no	nd/nd
Sd galectin 1	*Suberites domuncula*	Hadromerida (D)	22.1 (nd)	galactoside residues	nd	no	nd	nd/nd
Sd galectin 2	*Suberites domuncula*	Hadromerida (D)	35.0 (nd)	galactose	nd	Ca^2+^	no	nd/nd
**Sponge C-type lectins**								
AaL	*Aplysina archeri*	Verongida (D)	63.0 (4)	non reducing galactoside residues	nd	Ca^2+^/Mg^2+^	no	nd/nd
AlL	*Aplysina lacunosa*	Verongida (D)	63.0 (4)	non reducing galactoside residues	nd	Ca^2+^/Mg^2+^	no	nd/nd
AvL	*Aphrocallistes vastus*	Hexactinosida (H)	34.0 (1)	galactoside residues	nd	Ca^2+^	no	nd/nd
CvL	*Cliona varians*	Hadromerida (D)	114.0 (4)	galactose/sucrose	nd	Ca^2+^	yes	6.0–8.0/<60 °C
Lb MBL	*Lubomirskia baicalensis*	Haplosclerida (D)	13	mannose	nd	nd	nd	nd/nd
PsL	*Pellina semitubulosa*	Halichondrida (D)	200.0 (6)	galactose/arabinose	6.1	Ca^2+^	no	nd/nd
**Sponge tachylectine-like lectins**								
Ef lectin	*Ephydatia fluviatilis*	Haplosclerida (D)	24.0 (1)	nd	nd	no	no	nd/nd
Sd lectin	*Suberites domuncula*	Hadromerida (D)	27.0 (1)	lipopolysaccharides	nd	no	no	nd/nd
**Sponge F-type lectin**								
CcL	*Crambe crambe*	Poecilosclerida (D)	14.0 (1)	fucose	nd	no	no	nd/nd
**Unclassified sponge lectins**								
AcL I	*Axinella corrugata*	Halichondrida (D)	78.5 (6)	*N*-acetyl-derived residues	6.3	no	yes	6.5–8.5/<65 °C
AcL II	*Axinella corrugata*	Halichondrida (D)	80	galatose/chitin/fetuin/*N*-acetyl-derived residues	nd	nd	nd	2.0–6.0/<65 °C
ApaL I	*Aaptos papillata*	Hadromerida (D)	21.0 (2)	*N*-acetyl-d-glucosamine	42,131	nd	no	nd/nd
ApaL II	*Aaptos papillata*	Hadromerida (D)	16.0 (1)	*N*-acetyl-d-glucosamine/*N*-acetyl-d-galactosamine/sialic acid residues	3.4/5	nd	no	nd/nd
ApaL III	*Aaptos papillata*	Hadromerida (D)	16.0 (1)	*N*-acetyl-d-glucosamine/*N*-acetyl-d-galactosamine/sialic acid residues	3.4/5	nd	no	nd/nd
ApL I	*Axinella polypoides*	Halichondrida (D)	nd (2)	galactoside residues	3.9	no	yes^1^	nd/nd
ApL II	*Axinella polypoides*	Halichondrida (D)	nd	galactoside residues	nd	nd	nd	nd/nd
ApL III	*Axinella polypoides*	Halichondrida (D)	nd	galactoside residues	nd	nd	nd	nd/nd
ApL IV	*Axinella polypoides*	Halichondrida (D)	nd	hexuronic acids	nd	nd	nd	nd/nd
ApL V	*Axinella polypoides*	Halichondrida (D)	nd	galactoside residues	nd	nd	nd	nd/nd
CaL	*Cinachyrella apion*	Spirophorida (D)	124.0 (8)	lactose	nd	no	no	nd/nd
CalL	*Cinachyrella alloclada*	Spirophorida (D)	nd	galactoside residues	nd	nd	yes	nd/nd
CauL	*Craniella australiensis*	Spirophorida (D)	54.0 (3)	porcine stomach mucin/asialo-porcine stomach mucin	nd	no	yes	5.0–8.0/20–70 °C
CnL	*Chondrilla nucula*	Chondrosida (D)	70.0 (4)	galactose	nd	no	no	4.5–8.5/<60 °C
CtL	*Cinachyrella tenuiviolacea*	Spiroporida (D)	22	lactose	nd	nd	nd	nd/nd
DaL	*Desmapsamma anchorata*	nd	nd (2)	galactose	nd	nd	nd	nd/nd
Halilectin 1 (H-1)	*Haliclona caerulea*	Haplosclerida (D)	15.0 (1)	nd	nd	no	no	9/<70 °C
Halilectin 2 (H-2)	*Haliclona caerulea*	Haplosclerida (D)	27.0 (2)	porcine stomach mucin	nd	no	no	4.0–5.0/<80 °C
Halilectin 3 (H-3)	*Haliclona caerulea*	Haplosclerida (D)	nd (4)	porcine stomach mucin/*N*-acetyl-galactosamine	nd	no	no	4.0–7.0/<70 °C
HcL	*Haliclona cratera*	Haplosclerida (D)	nd	orosomucoid	8.6	no	no	4.6–10.2/56 °C
HL	*Haliclona sp.*	Haplosclerida (D)	24.0 (nd)	galactose/lactose	nd	nd	nd	nd/nd
HoL-1	*Halichondria okadai*	Halichondrida (D)	84.0 (4)	*N*-acetyl groups of *N*-acetyl-d-glucosamine or *N*-acetyl-d-galactosamine	4.5	no	yes ^1^	nd/<50 °C
HoL-2	*Halichondria okadai*	Halichondrida (D)	42.0 (1)	galactoside β1-4*N*-acetyl-d-glucosamine units	4.5	no	no	nd/<40 °C
HpL	*Halichondria panicea*	Halichondrida (D)	78.0 (4)	fetuin/galacturonic acid/glucuronic acid/polygalacturonic acid/fucose	nd	no	yes ^1^	7.2–9.5/<30 °C

nd: not determined; D: Demospongiae; H: Hexactinellidae; yes ^1^: intrachain disulfide loop involved in the binding activity of the lectin; pI: isoelectric point.

## 3. Sponge Lectin Purification, Sequencing and Expression

Lectins were formerly purified from sponge crude extracts [48,49,50]. However, with the emergence of molecular biology and genome sequencing, they were later identified directly from sponge expressed sequence tag (EST) libraries. In some cases, recombinant lectins were produced in heterologous expression systems [42,51,52].

### 3.1. Purification Strategies

Different purification protocols starting from sponge crude extracts were reported in the literature (summarized in Table 2). Briefly, sponge specimens were crushed and soaked from 2 h to overnight in a buffered aqueous solution, which usually consisted of Tris Buffered Saline (TBS), Phosphate Buffered Saline (PBS) or distillated water ranging from 4 to 20 °C at pH 7–8 [49,53,54]. This method creates an osmotic shock, which breaks the cell membranes and therefore easily liberates molecules from cytosol. Some protocols used calcium- and magnesium-free seawater (CMFSW) supplemented with 250 mM ethylenediaminetetraacetic acid (EDTA) [50,55,56] in order to maintain the seawater osmolarity and to prevent links between membrane proteins involved in sponge cell-cell adhesion [57]. A modified extraction process was sometimes applied by adding CaCl_2_ salts in the extraction buffer [58], since the carbohydrate-binding activity depends on the presence of Ca^2+^ ions.

Precipitation steps were sometimes performed in order to concentrate the lectin fractions. A Ca^2+^ precipitation can contribute to collect by centrifugation Ca^2+^-conjugated molecules and therefore to promote the enrichment of lectins structurally dependent of Ca^2+^ [56]. Some studies also implemented acetone precipitations in order to concentrate proteins from the extracts [59,60]. Dialysis was also performed in order to replace the extraction buffer with appropriate buffers for the following steps [61].

In general, at least two purification steps were necessary before the haemagglutination assay in order to select active lectin-containing fractions. In most cases, the first purification step involved affinity chromatography [53,54], according to the carbohydrate specificity of the lectin (Table 2). Due to the strong affinity of sponge lectins for galactoside residues, sepharose or lactose-conjugated matrices were often selected [61,62]. By using affinity chromatography with rabbit erythrocyte stroma followed by a gel filtration on Ultrogel AcA 44 column, the *Axinella corrugata* (George and Wilson, 1919) lectins-I and -II (AcL-I and -II) were purified [53,63]. Sometimes, a specific antibody directed against lectins was fixed onto a column in order to retain the lectins, as illustrated with the *Cinachyrella apion* (Uliczka, 1929) lectin (CaL), which was purified by using an antibody directed against the *Cliona varians* (Duchassaing and Michelotti 1864) lectin (CvL) [59,60]. Some lectins were also eluted from the affinity matrix using buffers selected according to different parameters, including substrate competition [58,62], disruption of weak interactions within the substrate-ligand complex [55], ionic force [54], pH stability [60], or chelation of ions (Ca^2+^) involved in the binding of the protein complex [59].

**Table 2:** Protocols of sponge lectin purification.

Species	Material	Extraction Buffer		Type I Purification	Column	Elution Buffer I	Type II Purification	Column	Elution Buffer II
*Aphrocallites vastus*	100 g (w) frozen	calcium- and magnesium-free seawater Ca^2+^ precipitation		gel filtration	Biogel P300 column	Calcium Magnesium free sea water	centrifugation on sucrose gradient		
*Axinella corrugata*		water/PBS pH 7.2		affinity chromatography	rabbit stroma-polyacrilamide gel	0.035 M NH_4_OH/0.154 M NaCl	gel filtration	Ultrogel—AcA 44	PBS
*Cinachyrella apion*		1:2 (*w*/*v*) in 0.05 M Tris-HCl pH 7.5 acetone precipitation		immunoaffinity chromatography	IgG anti CvL-Sepharose	0.050 M Tris-HCl pH 11	gel filtration	Superose 6 10/300	0.05 M Tris-HCl pH 7.5
*Cliona varians*		1:2 (*w*/*v*) in 0.05 M Tris-HCl pH 7.5 acetone precipitation		affinity chromatography	Sepharose CL 4B	0.05 M Tris-HCl/0.1 M EDTA pH 8	ion exchange	CM cellulose column	acetate buffered saline
*Craniella australiensis*	23 g (w)	1:10 in 0.9% NaCl dialysis against water		ion exchange	DEAE-Sephacel	gradient of NaCl in 0.010 Tris-HCL pH 7.4	gel filtration	Sephadex G-150	0.1 M PBS pH 7.4
*Geodia cydonium*		calcium- and magnesium-free sea water		affinity chromatography	lactose-divinylsulfone-agarose	PBS/0.05% Tween 20	precipitation with carbohydrates		
*Haliclona caerulea*		1:10 (w/v) in deionized water		affinity chromatography	glutaraldehyde-fixed human erythrocyte stroma-Sephadex G25	TBS/0.3% NH_4_OH pH 8.5	gel filtration		
*Haliclona cratera*	150 g (w)		TBS pH 7.5 polyvinylpolypyrrolidone/+protease inhibitors	affinity chromatography	CM Sepharose 4B CL	0.020 M phosphate buffer/1 M NaCl pH 7.5	gel filtration	Bio Gel P-100	TBS/152 mM NaCl
*Halichondria okadai*	200 g (w) frozen		1:10 (*w*/*v*) TBS/0.15 M NaCl/0.01 M protease inhibitor mix pH 7.4	affinity chromatography	lactosyl agarose	TBS/0.1 M lactose pH 7.4	gel filtration	Sephadex 75	TBS pH 7.4
*Halichondria panicea*	30 g (w)		1:3 (*w*/*v*) in calcium- and magnesium-free sea water/0.25 M EDTA/0.01 M 2-mercaptoethanol	affinity chromatography	Sepharose 4B				
*Pellina semitubulosa*	240 g (w)		1:1 (*w*/*v*) in 0.03 M Tris-HCl/2 mM CaCl_2_ pH 8.5	affinity chromatography	acid-treated Sepharose 6B	0.03 M Tris-HCl/0.002 M CaCl_2_/0.1 M lactose pH 7.5	gel filtration	Sephadex G-200	0.03 M Tris-HCl pH 7.5

w: wet weight; PBS: phosphate buffered saline; TBS: Tris buffered saline; EDTA: ethylene diamine tetraacetic acid.

In most cases, this first purification step was usually followed by gel filtration chromatography in order to separate lectins of different molecular masses [50,53,54]. By using this method, both *A. corrugata* lectin-I and -II (AcL-I and AcL-II) were purified from the sponge *A. corrugata*. Their relative molecular weights were estimated by gel filtration as 78.5 and 80 kDa, respectively [53,63]. Some lectins were precipitated by using carbohydrates conjugated to poly[*N*-(2-hydroxy-ethyl)acrylamide] after affinity chromatography [55].

Original protocols were also set up, as illustrated with the purification of the *C. varians* lectin. This latter was purified by affinity chromatography followed by ion exchange chromatography (carboxymethyl cellulose column) [59]. The purification of the *Craniella australiensis* (Carter, 1886) lectin (CauL) was performed by ion exchange chromatography (DEAE-Sephacel) followed by gel filtration [61].

The partial structural elucidation of lectins was usually performed using mass spectrometry [49,62,64] or Edman degradation [61,65].

### 3.2. Genome Screening and Lectin Production Strategies

The construction of sponge cDNA libraries is a complementary method to directly access to the lectin gene sequences. Therefore, cDNA libraries from the hexactinellid sponge *Aphrocallistes vastus* (Schulze, 1886) [56] and the demosponges *Cinachyrella* sp. [65], *Ephydatia fluviatilis* (Linnaeus, 1759) [42], *Geodia cydonium* (Jameson, 1811) [66], *Lubomirskia baicalensis* (Pallas, 1773) [52] and *Suberites domuncula* (Olivi, 1792) [51] were screened using degenerate primers [52,56] or EST-sequence libraries.

**Figure 1:**
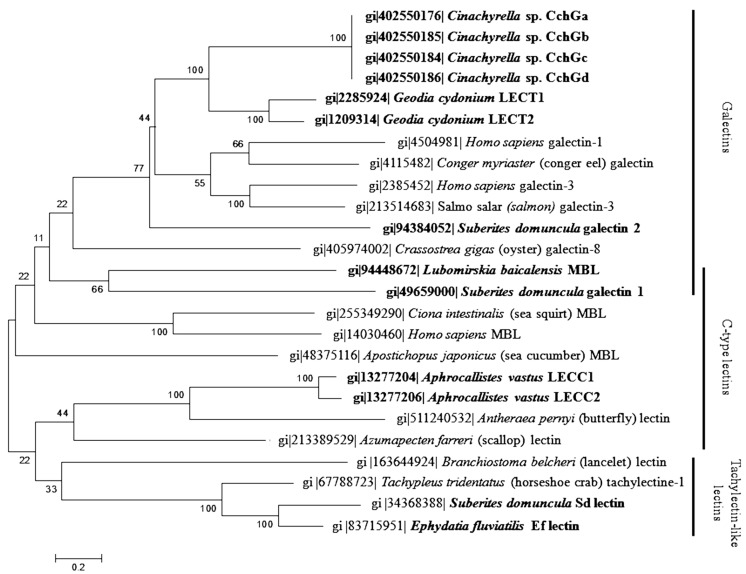
Neighbor-joining phylogenetic tree generated by analyzing protein sequences of sponge lectins from *Aphrocallistes vastus*, *Cinachyrella* sp., *Ephydatia fluviatilis*, *Geodia cydonium*, *Lubomirskia baicalensis*, *Suberites domuncula* (in bold) and animal lectin sequences from GenBank. Each entry is preceeded by its GenBank accession number. The tree was constructed using maximum composite likelihood and pairwise deletion. Percentage bootstrap values from 1000 re-samplings, indicated at the nodes, represent the level of confidence for the branches. Scale bar indicates an evolutionary distance of 0.2 amino acid substitutions per position in the sequence.

A phylogenetic alignment was implemented using the software MEGA 6 in order to compare protein sequences of sponge lectins to other animal lectins from GenBank. A neighbor-joining phylogenetic tree from this alignment using 1000 bootstrap repeats is shown in Figure 1. The results revealed that the studied sponge lectins have evolved from different proteins, already present in the common ancestor of Eumetazoa and Porifera. Sponge galectin, C-type and tachylectin-like lectin genes are more closely related to other animal lectin genes than to sponge lectin genes, except for the sponge galectin genes from *G. cydonium* and *Cinachyrella* sp., which are phylogenetically very similar. Both sponge galectin genes are related to vertebrate galectin genes such as the human galectin 1 and 3, the salmon galectin 3 and the conger eel galectin. Surprisingly, both *S. domuncula* galectin 1 and 2 genes are phylogenetically separated and more related to *Crassostrea gigas* galectin-8. The *S. domuncula* galectin-2 gene was found to be closely related to the *L. baicalensis* MBL gene, encoding for a C-type lectin. The MBL, including *L. baicalensis* MBL, and the other sponge C-type lectins, including *A. vastus* lectin, are phylogenetically separated, suggesting that they have evolved from different proteins. The sponge tachylectin-like lectins from *S. domuncula* and *E. fluviatilis*, named *S. domuncula* lectin and *E. fluviatilis* lectin, respectively, form with tachylectin-1 and *Branchiostoma belcheri* lectin a phylogenetically conserved group that have evolved from an ancestral protein. The C closely related-type lectin from *A. vastus* belongs to this group, suggesting that members of this group may have also differently evolved.

In some studies, sponge lectins were produced in *E. coli* expression system using either glutathione-*S*-transferase gene containing plasmid pET-41a (+) [51] or pGEX2T [57,67] or a *N*-terminal histidine tag-containing plasmide (pEcoli-Nterm-6 × HN Vector). In the latter case, the lectin was purified through a nickel column [65]. These expression strategies delivered a large amount of recombinant proteins for studying their biotechnological potential [63] and/or their physiological roles within sponges [51].

## 4. Biochemical Properties of Sponge Lectins

To the best of our knowledge, 39 different lectins were characterized from the phylum Porifera so far (Table 1). All of them were isolated from demosponges, except the C-type lectin, obtained from the hexactinellid sponge *A. vastus* (Schulze, 1886) [56]. The relative molecular masses of lectins were usually determined by gel filtration under native conditions and the relative molecular masses of their subunits by SDS-PAGE under denaturating conditions. Therefore, the molecular weights of the subunits determined by SDS-PAGE do not always fit with the total relative molecular masses of the lectins, the steric conformation of the proteins being abolished under denaturating conditions. In this review, the reported molecular masses of the different lectins were calculated from the results of gel filtration chromatography, while the molecular masses of their subunits were obtained by SDS-PAGE.

### 4.1. Galectins

Six sponge lectins were identified as galectins: the *Geodia cydonium* galectin, the *Suberites domuncula* galectins 1 and 2, the *Halichondria okadai* galectin 30 and the *Cinachyrella* sp. galectins 1 and 2.

The *G. cydonium* galectin (GCG) was the most studied lectin among the Porifera phylum. GCG was firstly isolated from specimens collected in the Mediterranean Sea by using an affinity chromatography coupling hog A+ blood group substances with Sepharose 4B. This lectin of 60 kDa turned out to be a tetramer with 15 kDa subunits and displayed an isoelectric point (pI) of 4.4. GCG showed to form a multimeric complex in presence of Ca^2+^, but the link between the lectin and the carbohydrates was not ion-dependent [57]. From a cDNA library obtained from the mRNA of the *G. cydonium* sponge, two cDNA clones were obtained for GCG, encoding two different GCG polypeptides LECT1 and LECT2 with deduced molecular masses of 15,313 kDa and 15,433 kDa, respectively [66,68,69,70,71]. Later, it was demonstrated that these two cDNAs were not derived from different genes and that three 13, 15 and 16 kDa monomers (GCG subunits) corresponded to alternative splicing variants of the same LECT1 gene. GCG shared characteristics from both prototype and chimera-type lectins [72]. The latter gene also displayed polymorphisms located in the carbohydrate recognition domain. This genetic variation did not concern amino acids involved in the interactions with the carbohydrates. In addition, investigations of the cDNA sequence of the galectin protein precursor revealed that the *N*-terminal region of the protein might act as a putative transmembrane segment. Following this sequence, an *N*-terminal secretion signal cleavage site was identified. It was postulated that this sequence probably recruited protease to get the soluble form of GCG. This feature is specific to this galectin because other animal galectins are synthesized without this signal sequence [73]. Analyses of soluble and membrane-containing fractions of *G. cydonium* revealed that GCG was present in both fractions. The mechanism of the protein maturation and the function of both soluble and membrane-binding galectins remained obscure. The *N*-terminal region of GCG soluble form contained also a protein domain, which is usually involved in the binding to prokaryotic membrane lipids [57,74]. Intriguingly, by using cytochemical staining of carcinoma cells, it was reported that the nuclear binding of the *G. cydonium* galectin was mediated by protein-protein interactions with a 56 kDa nucleoplasmic protein (np56) [55].

*S. domuncula* galectins 1 and 2 (Sd galectins 1 and 2) were identified *in silico* from a *S. domuncula* cDNA library and were produced *in vitro* by using a bacterial heterologous system. The deduced amino acid sequence of the two cDNA clones displayed a molecular mass of 22.1 and 35 kDa, respectively. The galactoseceramide Sdgal-1 isolated from *S. domuncula* was found to inhibit the carbohydrates-binding activity of the Sd galectin 1. The Sd galectin 2 underwent self-association in the presence of Ca^2+^. The primary structure of this galectin consisted of two galactose-binding sites and a hydrophobic domain at the *N*-terminal region. Its *C*-terminal hydrophobic domain showed to interact directly with α silicatein, an enzyme involved in the spicule formation in siliceous sponges. Therefore, the Sd galectin 1 belongs to prototype galectin, whereas the Sd galectin 2 can be classified as a member of the tandem repeat family of galectins [51,67,75].

The order Halichondrida afforded the *H. okadai* galectin 30 (HoL 30) from the Japanese sponge *H. okadai*, by using an affinity chromatography. HoL 30 displayed a molecular mass of 60 kDa (two subunits of 30 kDa) and a pI of 6.7. Its agglutination activity on rabbit and human erythrocytes was inhibited by different sugars including galactoside β1-3 *N*-acetyl-glucosamine (Galβ1-3GlcNac) and galactoside β1-4 *N*-acetyl-glucosamine (Galβ1-4GlcNac) motifs. Moreover, HoL 30 showed a high homology with the amino acid sequences of the *G. cydonium* galectin [62].

By using affinity chromatography, both *Cinachyrella* sp. galectins 1 and 2 (CchG 1 and CchG 2) galectin prototypes were purified from *Cinachyrella* sp. sponges collected off Japanese coasts (Okinawa). Six cDNAs encoding CchGs with putative chimera were found. However, peptide sequencing revealed at least two isoforms with very similar galactoside-binding domains, which showed to be tolerant to high temperatures (100 °C). According to the authors, these galectins aggregated to create a stable tetramer [65]. CchG 1 was the first three-dimensional structure of a sponge lectin determined by X-ray crystallography. This lectin demonstrated to be formed by two sequences of five antiparallel β-sheets paired face-to-face through a hydrophobic core. Therefore, CchG 1 consisted of a rigid toroidal-shaped “donut-hole” tetrameric arrangement, whose final structure was based on the presence of canonical dimers. This dimer-of-dimers interface showed to be mediated by a novel structure that was stabilized by the packing of pairs of vicinal disulfide bonds between adjacent cysteines [76].

### 4.2. C-Type Lectins

Six lectins were classified as C-type lectin members due to their carbohydrate-binding activity depending of the presence of Ca^2+^ or Mg^2+^: the *Pellina semitubulosa* (Lieberkühn, 1859), the *Aplysina archeri* (Higgin, 1875) and the *Aplysina lacunosa* (Lamarck, 1814), as well as the *Aphrocallites vastus*, the *Cliona varians* and the *Lubomirskia baicalensis* MBL lectins.

The marine sponge *P. semitubulosa*, collected in the Adriatic Sea, provided a 200 kDa-lectin (PsL), composed by hexameric glycoproteins of 34 kDa, which were linked by disulfide bridges. Its binding activity on human, sheep and rabbit erythrocytes was inhibited by galactose and arabinose. The protein had a pI of 6.1 and its binding activity was stable up to 56 °C [58].

Two biochemically similar lectins, the *Aplysina archeri* (AaL) and *Aplysina lacunosa* (AlL) lectins, were isolated from *A. archeri* and *A. lacunosa*, respectively, collected along Venezuelan coasts. Both lectins, purified by using affinity chromatography on *p*-aminobenzyl-β-1-thiogalactopyranoside-agarose followed by gel filtration chromatography, exhibited a molecular mass of 63 kDa. Both lectins were tetramers of 16 kDa-subunits. Their agglutination activity towards hamster and rabbit erythrocytes was dependent of divalent Ca^2+^/Mg^2+^ cations. Experimental data revealed that AaL bound preferentially non-reducing β-linked galactoside residues [77].

The *C. varians* lectin (CvL), purified using a Sepharose CL 4B affinity chromatography, revealed a molecular mass of 114 kDa by using gel filtration chromatography. However, the SDS-PAGE analysis demonstrated that the lectin was composed of four 28 kDa-subunits linked by disulfide bridges. The agglutination activity of CvL towards human erythrocytes was inhibited by galactose and sucrose. The protein was stable at a pH ranging from 6 to 8 and at a temperature lower than 50 °C [59].

The only lectin purified from a hexactinellid sponge was the *A. vastus* lectin (AvL). This lectin was isolated by using Ca^2+^ precipitation and Biogel P300 gel filtration chromatography. This C-type lectin was a glycosylated 34 kDa-polypeptide that revealed to preferentially bind galactose in the presence of Ca^2+^ ions. By using degenerated primers against a cDNA library, two cDNA sequences of C-type lectins were then identified from this sponge coding for two polypeptides with a calculated molecular mass of 22,022 and 22,064 kDa, respectively. They displayed high similarities with C-type lectins from higher Metazoa. Their primary structure contained conserved sites for disulfide bridges, Ca^2+^-binding sites, a galactose-binding domain and a hydrophobic *N*-terminal domain [56].

The *L. baicalensis* MBL (Lb MBL) was identified from a *L. baicalensis* cDNA library [52]. The protein had a predicted molecular mass of 13 kDa, according to the open reading frame of the cDNA, with sequence similarities with mannose-binding lectins from other animals [52].

### 4.3. Tachylectin-Like Lectins

Two tachylectin-like lectins, named *E. fluviatilis* lectin (Ef lectin) and *S. domuncula* lectin (Sd lectin), were identified from *S. domuncula* and *E. fluviatilis*, respectively.

Sd lectin is a monomer of 27 kDa isolated from specimens collected in the Mediterranean Sea using an affinity chromatography. This lectin showed to bind LPS of Gram negative bacteria and to share high sequence similarities with the horseshoe crab *Tachypleus trunculus* lectin [40]. The cDNA sequence of Sd lectin displayed a predicted transmembrane region, suggesting an association with the membrane [41].

Ef lectin was identified from an EST library of the freshwater sponge *E. fluviatilis*. This polypeptide with a predicted molecular mass of 24 kDa is closely related to both Sd lectin [41] and tachylectin1/L6 lectin from *T. tridentatus* [40]. The deduced amino acid sequence revealed six tachylectin repeats [42].

### 4.4. F-Type Lectin

The *Crambe crambe* (Schmidt, 1862) lectin (CcL) exhibited a specific affinity for fucose. CcL lectin was purified using an affinity chromatography on a Sepharose column followed by gel filtration chromatography. The agglutination activity of CcL towards sheep and human erythrocytes was only inhibited by fucose. Its activity was stable at a pH range from 4.5 to 8.5 and at temperatures lower than 60 °C for 1 h [78]. This lectin displayed a lot of basic amino acids in its protein sequence, what is unusual for a sponge lectin [54].

### 4.5. Unclassified Sponge Lectins

Twenty-four sponge lectins could not match with the above reported classes of lectins. Nevertheless, using their binding specificity and characteristics, we propose to group them into new lectin groups, including lectins containing intrachain disulfide bridges, mucin-binding, and *N*-actyl-d-glucosamine-/*N*-actyl-d-galactosamine-binding lectins.

#### 4.5.1. Intrachain Disulfide Bridge-Containing Lectins

Four lectins exhibited a disulfide loop within their subunits, potentially involved in the carbohydrate-binding activity: the *Axinella polypoides* (Schmidt, 1862) lectins I and II, the *Halichondria panicea* lectin and the *Halichondria okadai* lectin 1.

The *A. polypoides* lectins I and II (ApL I and II) were initially purified by using galactose-binding affinity chromatography. The mitogenic activity of ApL I on human purified lymphocytes was inhibited by galactose, fucose and 2-deoxy-galactose [45,79]. ApL I was firstly classified into the (QxW)_3_ lectin group, in which lectins normally present three QxW motifs in their protein sequence [80]. Later, this classification was modified with the identification of the *A. polypoides* lectin II, which exhibited the same structural characteristics. Both lectins have only one QxW motif and their intrachain disulfide loop showed to play a crucial role in the binding activity onto carbohydrates [64].

The *H. panicea* lectin (HpL), purified by using an affinity chromatography on Sepharose 4B followed by gel filtration chromatography on Biogel P300 column, had a molecular mass of 78 kDa and was formed by four subunits of 21 kDa. The haemagglutination inhibitory activity by thiol groups indicated that the disulfide bridges were involved in the binding activity. This agglutination was also inhibited by competition with fetuin, galacturonic acid, glucuronic acid, polygalacturonic acid and fucose. The protein was thermosensitive up to 30 °C and showed optimal binding activity ranging from pH 7.2 to 9.5. HpL lectin showed to bind galacturonic acid, glucuronic acid and fucose [50].

The *H. okadai* lectin-1 (HoL-1) exhibited the same intramolecular disulfide linkage, as suggested by the presence of a diffuse protein band visualized in SDS-PAGE in the absence of reducing agents. By using affinity chromatography and gel filtration, HoL-1 was purified with a predicted molecular mass of 84 kDa (four subunits of 21 kDa) and an isoelectric point at 4.5. The haemagglutination activity of HoL-1 was specifically inhibited by *N*-acetyl groups-containing sugars [81].

#### 4.5.2. Mucin-Binding Lectins

Among unclassified sponge lectins, the *C. australiensis* lectin and the *Haliclona caerulea* (Hechtel, 1965) Halilectins-2 and -3 were found to bind specifically mucin and/or orosomucin.

The *C. australiensis* lectin (CauL) was isolated by using an ion-exchange chromatography and gel filtration chromatography. CauL displayed a molecular mass of 54 kDa, due to its three subunits of 18 kDa. This protein was detected thanks to its capacity to agglutinate human, mouse, sheep, rabbit and chicken erythrocytes. This agglutination was only inhibited by porcine stomach mucin and asialo-porcine stomach mucin, suggesting its putative capacity to bind cell membrane. Its activity was stable from 20 to 70 °C and at a pH ranging from 5 to 8 [61].

The Halilectin-2 lectin (H-2) was purified using human A-type erythrocyte stroma fixed onto a Sephadex column bio-guided by its haemagglutination activity against rabbit erythrocytes [49]. With a molecular mass of 27 kDa (a dimer of 15 kDa-subunits linked by a weak interaction), H-2 displayed the highest activity in the basic range (pH 9) and at temperatures lower than 80 °C. H-2 was found to preferentially bind orosomucin and porcine stomach mucin. In comparison with protein sequences of other animal lectins, H-2 revealed an original primary structure. Nevertheless, H-2 displayed similarities with a contig from *Amphimedon queenslandica* (Hooper and van Soest, 2006) DNA library [49,82].

Halilectin-3 lectin (H-3) was purified by using a combination of hydrophobic interaction chromatography on a Phenyl-Sepharose 6B column and ion exchange chromatography. The lectin linked preferentially porcine stomach mucin and *N*-acetylgalactosamine. Its activity was stable from pH 4 to 7 and up to 70 °C. Native gel electrophoresis and gel filtration showed H-3 had a quaternary structure organized in a heterotetramer. Mass spectrometry analysis and *de novo* sequencing revealed that the protein displayed four subunits, resulting of a combination of alpha and beta subunits. The alpha subunit was a glycosylated polypeptide of 16 kDa, which exhibited a pyroglutamic acid residue at the *N*-terminus. The beta subunit showed a molecular mass of 11 kDa and also presented glycosylation. H-3 lectin was a blue protein presenting the rare property to bind a natural chromophore [83].

#### 4.5.3. *N*-Acetyl-d-glucosamine-/*N*-Acetyl-d-galactosamine-Binding Lectins

Fifteen lectins of this group demonstrated to bind either *N*-acetyl-d-glucosamine or *N*-acetyl-d-galactosamine residues-containing carbohydrates. Some of them may be related to galectins but a lack of data prevented their classification. This group gathers the *Aaptos papillata* (Keller, 1980) lectins I, II and III, the *A. corrugata* (George & Wilson, 1919) lectins I and II, the *A. polypoides* lectins III, IV and V, as well as the *Cinachyrella tenuiviolacea* (Pulitzer-Finali, 1982) lectin, the *Haliclona* sp. lectin, the *Cinachyrella alloclada* (Uliczka, 1929), the *Chrondrilla nucula* (Schmidt, 1862), the *Desmapsamma anchorata* (Carter, 1882), the *Halichondria okadai*-2 and the *Cinachyrella apion* lectins.

Three lectins (ApaL I, II and III) were isolated from the sponge *A. papillata* using affinity chromatography with polyleucyl blood group A + H substance from hog stomach as an adsorbent. They displayed molecular masses of 21 (two subunits of 12 kDa), 16 and 16 kDa for ApaL I, II and III, respectively. ApaL I binding activity was optimal at pH range 5–7 during electrofocusing, whereas the ApaL II and III binding activities were optimal at pH range 3.4–5. ApaL I revealed to bind non-reducing *N*-acetyl-d-glucosamine with a high affinity similarly to ApaL II and III, but this latter also demonstrated to bind *N*-acetyl-d-galactosamine and sialic acid residues [48].

Both *A. corrugata* lectins I and II (AcL I and II) were purified by affinity chromatography with rabbit stroma-polyacrylamide gel column followed by a gel filtration. AcL I was eluted using PBS and the second one was subsequently obtained with water. AcL I and II displayed a molecular mass of 78.5 kDa (four subunits of 13.9 kDa) and 80 kDa, respectively, determined by gel filtration chromatography. AcL I subunits were linked by disulphide bridges, forming a polymeric structure with a pI of 6.3. AcL I showed to link with high affinity *N*-acetylated carbohydrates, such as *N*-acetyl-d-galactosamine, but not galactose, whereas AcL II revealed to preferentially bind galactose, chitin, fetuin and *N*-acetyl-derivated saccharides but not *N*-acetyl-d-galactosamine. The haemagglutination activity of AcL I on rabbit, goat and dog erythrocytes and of AcL II on rabbit erythrocytes was independent of Ca^2+^, Mg^2+^ and Mn^2+^. The binding activity of AcL I was optimal at pH 7–8, whereas AcL II displayed a better stability at a pH range of 2–6 [53,63].

Regarding the other lectins, little is known about their biochemical characteristics. Both *A. polypoides* lectins III and V (ApL III and V) were found to specifically bind galactoside saccharides and the *A. polypoides* lectin IV (ApL IV) was found to bind more specifically hexuronic acids [79]. The *C. tenuiviolacea* lectin (CtL) (22 kDa) was purified by using a gel filtration chromatography followed by affinity chromatography. The agglutination activity of CtL towards human erythrocytes of ABO groups was inhibited by lactose, suggesting a potential specificity for β1-4-linked galactose residues. From an unidentified marine sponge of the *Haliclona genus*, the *Haliclona* lectin (HL) was isolated, revealing a molecular mass of 22 kDa, as estimated by SDS electrophoresis. Among all tested sugars, haemagglutination activity of HL against human erythrocytes was only inhibited by lactose [46]. The *C. alloclada* lectin (CalL) was isolated by using an affinity chromatography. The carbohydrates which carry galactose and non-reducing galactosyl groups showed to inhibit its agglutination activity on human erythrocytes [84]. The *C. nucula* lectin (CnL) exhibited a molecular mass of 70 kDa, consisting of four subunits of 15.6 kDa, and a pI of 4.5. It was purified by using an affinity chromatography followed by a haemagglutination assay with human erythrocytes (A, B, O). It displayed a specific binding activity for galactose [85]. By using affinity chromatography on a raffinose matrix bio-guided by haemagglutination activity on human erythrocytes, a galactose-specific lectin was obtained from specimens collected along Brazilian coasts and identified as *D. anchorata* lectin (DaL). DaL was composed of two subunits of 18 and 36 kDa [86]. The *H. okadai* lectin-2 (HoL-2) was identified by using an affinity chromatography. HoL-2 displayed a molecular mass of 42 kDa and a binding specificity for Galβ1-4GlcNac unit-containing sugars [80]. The *C. apion* lectin (CaL) was purified from specimens collected along a Brazilian coast using an affinity chromatography with IgG anti-*C. varians* lectin and its haemagglutination was tested towards human erythrocytes [60]. This lectin of 124 kDa was found to be composed by eight subunits of 15.5 kDa, linked by hydrophobic interactions. The binding activity of CaL was inhibited by lactose. The protein was stable from 0 to 60 °C. The *N*-terminal sequencing of the CaL revealed similarities with the silicatein from *S. domuncula* [60,75].

#### 4.5.4. Miscellaneous

The *H. caerulea* Halilectin-1 (H-1) lectin is the only unclassifiable lectin since its carbohydrate specificity could not be determined. H-1 was purified by using human A-type erythrocyte stroma fixed onto a Sephadex column bio-guided by its haemagglutining activity against rabbit erythrocytes. H-1 had a molecular mass of 15 kDa. Its binding activity was optimal from acid pH 4–5 up to 50 °C [49].

## 5. Physiological Roles of Sponge Lectins

Several studies have demonstrated that sponge lectins display different physiological roles such as involvement in the morphogenesis and cell interactions, spiculogenesis, defense of sponges or communication with associated microorganisms (Table 3).

### 5.1. Morphogenesis and Cell Interaction

By their properties to bind carbohydrates, lectins have showed to create molecular bridges between molecules and activate cell pathways in order to trigger different cell responses. The *A. polypoides* lectins I and II showed to be involved in production of spongin in the sponge mesohyl. Immunohistological studies demonstrated that these lectins were stored in spherule cells and within the spongin fibers of the sponge. Due to their presence around the sites of spongin production, the spherule cells were assumed to be involved in the spongin fiber production. ApL I and II, also detected in vesicles within the spherule cells, might participate to the assembly of the fibers [87].

The involvement of the *S. domuncula* galectin 1 in the formation of aqueous channels was demonstrated within the sponge *S. domuncula.* This lectin, beforehand produced as a recombinant protein in *E. coli*, was incorporated into a gel matrix, on which primmorphs (3D cell culture) were cultivated. These latter developed channel-like structures, that were very similar to these produced by gemmules during their hatching. This morphogenesis process was inhibited by the natural competitor, Sdgal-1, a β-galactoceramide isolated from *S. domuncula* [51]. Animal lectins had already shown their involvement in cell differentiation processes, especially at the embryonic stage, as illustrated with the participation of both galectin 1 and 3, secreted by numerous vertebrates, in fetal development, cell migration, tissue modeling. Both galectins were also supposed to be involved in cell-cell and cell-extracellular matrix interaction as adhesion modulator thanks to their ability to bind carbohydrates from extracellular matrix and cell surface molecules [88]. Furthermore, the ascidian *Didemnum ternatanum* lectin showed to promote cell agglutination, growth, as well as the cell differentiation at the gastrula stage of the embryonic mussel and sea urchin cells [89].

**Figure 2:**
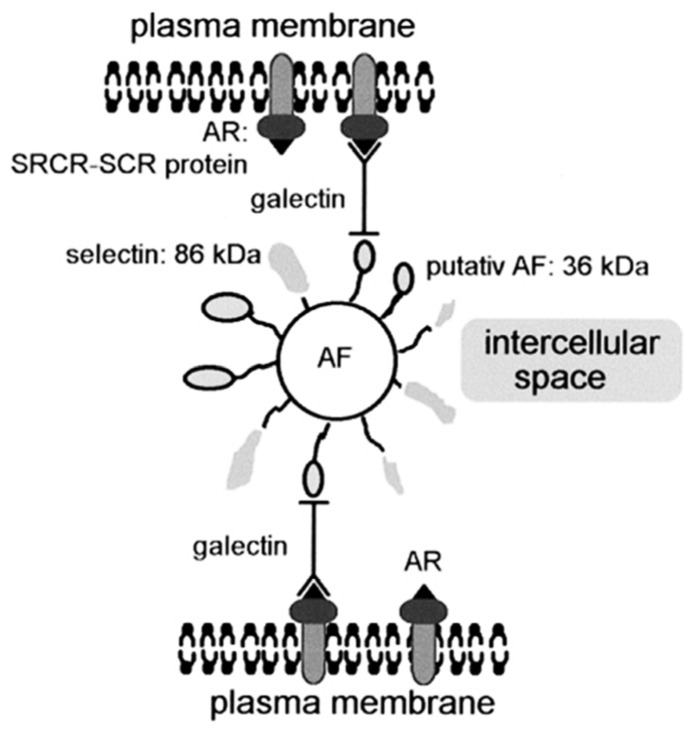
Schematic representation of the aggregation factor (AF)-mediated cell recognition in *G. cydonium* presented by Schütze *et al.* [92]. The aggregation receptor (AR) is inserted into the plasma membrane. The galectin might bind to the putative AF either directly, as represented on the scheme, or after dimerization in the presence of Ca^2+^. The galectin probably links the AR (a molecule composed of scavenger receptor cysteine-rich (SRCR) domains and short consensus repeats (SCRs)) via the putative AF protein to the AF core structure. A second putative protein, the selectin, is likely bound to the AF.

Sponge lectins also proved to mediate the sponge cell-cell interactions. The most studied lectin in this physiological context was the *G. cydonium* galectin. GCG was involved in a complex system linking sponge cells via an aggregation factor (AF), which was formed by selectin-related molecules (86 kDa) and aggregation factor proteins (36 kDa) [90,91,92]. This complex was linked to an aggregation receptor (scavenger receptor cysteine-rich/short consensus repeat), localized on the membrane of the sponge cells [93]. It has been hypothesized that GCG could link the AF to the membrane aggregation receptor of the carbohydrate binding domains either by a dimer of the *G. cydonium* galectin in the presence of Ca^2+^ or by another protein domain of one *G. cydonium* galectin monomer [57]. This latter protein domain was recognized by the aggregation receptor, as illustrated in Figure 2 [92].

A similar scheme occurred in the hexactinellid sponge *A. vastus*. The occurrence of an AF during the cell agglutination process was previously reported [94]. This cell agglutination demonstrated to rely on the presence of Ca^2+^. Biochemical analyses of the AF revealed that this factor was a lectin with a hydrophobic *N*-terminal domain. The authors proposed that this lectin was able to bind onto the cell membrane via the *N*-terminal region of the protein. It also interacted with carbohydrates on the surface of other cells/syncytia through its C-type lectin protein domain [56].

The role of lectins in cell adhesion was already demonstrated for other organisms. In vertebrates, this role is played by selectins, which are membrane-bound lectins, localized on the surface of endothelial (E-selectins) and immune (l-selectins) cells. These proteins display lectin-like domains, which mediate the initial adhesion of immune cells in order to activate the transient contact necessary for cells to move onto the vessel wall [95]. The endothelial leukocyte adhesion molecule-1 (ELAM-1), secreted by blood vessel cells, demonstrated to mediate cell adhesion of leukocytes via recognition of a specific carbohydrate ligand found on neutrophil cells [96]. Similarly, in invertebrates, this role is played by secreted lectins. A 18-kDa lectin-like aggregation factor, purified from the horseshoe crab *Limulus polyphemus*, revealed to agglutinate horse erythrocytes and to aggregate *Limulus* amebocytes [97]. The C-type Zhikong scallop *Chlamys farreri* lectin-2 also revealed its ability to bind the surface of scallop hemocytes and to initiate the hemocytes recruitment in order to enhance the immune encapsulation process [98]. Such roles in innate immunity have been also demonstrated by the *Drosophila melanogaster* galectins 2 and 3 and the bay scallop *Argopecten irradians* lectin AiCTL-9, which have shown to bind hemocytes and to enhance the cellular encapsulation [99,100].

The sponge lectins, localized in the mesohyl, have demonstrated to promote the tissue organization and cell-cell adhesion. This role in cell adhesion seems to be conserved in the immune cellular responses in vertebrates as well as in invertebrates. Moreover, animal lectins showed to influence the cell differentiation processes, especially at the embryonic stage. This embryonic role is similar to the role of galectin 1 in the sponge *S. domuncula* during the gemmule hatching.

### 5.2. Biomineralization and Spiculogenesis

In some cases, lectins showed to be involved in the formation of sponge spicules, as reported for both sponges *S. domuncula* and *L. baicalensis*.

**Figure 3:**
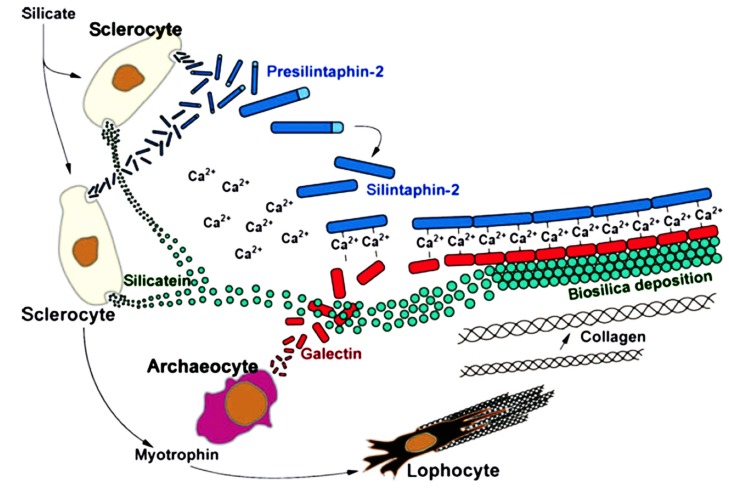
Cellular and molecular interactions during spiculogenesis in *S. domuncula* involving different cell types and their secreted molecules [75]. Sclerocytes and archeocytes synthesize three main components which allow the growth of spicules (pre-silintaphin 2, silicatein and galectin 2). Collagen fibers are synthesized by lophocytes stimulated the secretion of myotrophin by sclerocytes.

The *S. domuncula* galectin 2 displayed two galactose-binding sites and a hydrophobic *N*-terminal region, which mediates the polymerisation in presence of calcium. The Sd galectin 2 was detected in the axial canal and on the surface of the spicules, directly interacting with silicatein through its *C*-terminal region, thus enhancing 2-fold the *in vitro* activity of the enzyme. Experiments demonstrated that the silicatein interactor, silintaphin-2, provided Ca^2+^ ions *in situ* to Sd galectin 2 proteins and promoted the interaction between the lectin and collagen molecules, as well as the formation of a gel-like scaffold around the growing spicules, as summarized in Figure 3. The Sd galectin 2 scaffold was used as a platform for silicatein molecules in order to polymerize orthosilicate into a silica layer around the spicule [67,101]. The Sd galectin 2, in presence of Ca^2+^, also provided a fluid/stable matrix for transporting metabolites and water. It has been postulated that water molecules, formed during biosilica synthesis, were trapped by this matrix and then imported into cells. This process contributes to the hardening of the spicules [75].

The MBL from the freshwater sponge *L. baicalensis* was detected around the spicules forming organic coats. Lb MBL and silicatein expression were variable along the apical-basal axis of the spicules. Their transcripts in the tissues surrounding the spicule were in high abundance in the top module of the branch, whereas only small amounts of transcripts were detected at the base of the branch. Their expression may be controlled by the mago nashi factor [52].

This proteinaceous network created by sponge lectins was also reported in other biomineralized structures, including egg and mollusk shells. Lectins are also able to modulate the nucleation of calcium carbonate crystals in these organisms. The ostrich *Struthio camelus* eggshell matrix exhibited two C-type lectins, struthiocalcin-1 and -2, which were able to form aggregates *in vitro*, enhancing the growth of calcite crystals during mineralization [102]. Similar *in vitro* behaviours were observed with the lectin ansocalcin, extracted from the goose eggshell matrix [103]. Lectins were also extracted from mollusk shells, as illustrated with the perlucin and perlustrin C-type lectins from the abalone *Haliotis laevigata*. Perlucin was supposed to nucleate and to bind calcium carbonate crystals, as well as mediating the interaction between the chitin and the aragonite layer [104,105]. The wing oyster *Pteria penguin* lectins PPL2A and PPL2B demonstrated to participate in the biomineralization of the shell using knockdown experiment at the larval stage [106]. In contrast, the acorn barnacle *Megabalanus rosa* C-type lectins BRA-1, -2, -3 inhibited the nucleation and the growth of calcium carbonate crystals, thus modulating the shape and size of of crystals [107].

Similarly to other organisms, sponge lectins provide a good scaffold for the assembly of silicatein molecules and showed to promote the formation and the hardening of biosilica onto growing spicules.

### 5.3. Host Defense

Due to antimicrobial activities towards prokaryotes and cytotoxic activities against eukaryotic parasites, some sponge lectins demonstrated to be involved in the sponge defense.

Both *C. varians* and *S. domuncula* lectins displayed a large spectrum of antibacterial activities. The *C. varians* lectin revealed to inhibit the proliferation of Gram positive bacteria, whose the cell wall is a peptidoglycan layer, whereas the *S. domuncula* lectin preferentially showed an antibacterial activity against Gram negative bacteria, binding specifically the LPS of bacteria. Sd lectin was overexpressed in response to a LPS treatment [41,59]. These particularities confirmed the assumption that sponges were able to discriminate between Gram-negative and Gram-positive bacteria and to settle a differential response according to the different types of bacteria [108,109,110]. Thanks to their ability to recognize different carbohydrates, sponge lectins can participate to the defense against bacteria.

Only a few data are available regarding sponge viruses. The *C. nucula* lectin showed to slow down the development of HIV-I virus by increasing the expression of (2′-5′) oligoadenylate synthase in infected human cells [85]. Virus particles have not yet been clearly detected in sponges, but an antiviral defense system seems to exist in sponges including potential molecules of lectin-type [111].

The *A. corrugata* lectin II has been shown to specifically bind chitin, which is composed of polymers of *N*-acetyl-glucosamine (GlcNac) [63]. According to the authors, this lectin may be related to the defensive function of sponge against fungal parasites. This polymer was highly present in the cell wall of fungi. Sponges already demonstrated to display a defense systeme against fungi, as illustrated by the fibrinogen-like molecule and the epidermal growth factor, which were produced in *S. domuncula* in response to a fungal cell wall molecule, the (1→3)-β-d-glucans, recognized by a specific tyrosine kinase receptor [112].

Some lectins have also revealed to protect sponges against predation from unicellular protozoans and marine animals. Some sponge lectins showed agglutination and cytotoxic activities against protozoans and crustaceans. Both *C. apion* and *C. nucula* lectins exhibited agglutination activity towards *Leishmania chagasi* [59,60]. The *H. caerulea* Halilectin-1 and -2 and the *A. corrugata* lectin I showed cytotoxic activity towards *Artemia nauplii* and *Artemia salina* [49,63]. These *in vitro* activities indicate that these sponge lectins might play a role in sponge defense against predators. As lectins demonstrated to specifically bind targeted carbohydrates, the cytotoxic effect of these lectins could be directed against specific eukaryote parasites.

Defense roles of lectins have been already widely investigated in plants as well as in animals because of their cytotoxic effects. The plant lectin abrin from the rosary pea was the first lectin recognized as a defense protein [113]. Since this work, plant lectins have demonstrated deleterious activities against a large panel of invaders including bacteria, fungi and predatory animals such as insects [114,115,116]. In vertebrates, these defense roles are mainly played by collectins and ficolins, which showed to bind carbohydrates from microorganisms. However, if they do not directly kill microorganisms, they are able to promote the recruitment of immune cells (opsonin), as illustrated with the SP-A human surfactant protein and the l-ficolin [117,118,119]. Similarly, in invertebrates, the MBP-CfC1qDC-2 collectin-related protein, isolated from the scallop *Chlamys farreri*, can bind numerous pathogen-associated molecular patterns (PAMP), such as LPS, peptidoglycan (PGN), β-glucan, mannan, as well as different microorganisms, including Gram-negative and Gram-positive bacteria as well as yeasts [120]. The *Crassostrea gigas* C1q complement-related protein CgC1qDC-1 revealed to bind LPS and was supposed to attract immune cells, promoting the phagocytosis of Gram-negative bacteria [121]. In addition to their opsonin role, invertebrate lectins also displayed antimicrobial activities on pathogens, as illustrated with the Manila clam *Ruditapes philippinarum* MCL-4 and the Chinese shrimp *Fenneropenaeus chinensis* lectins [122,123].

Sponge defense lectins displayed antimicrobial and cytotoxic activities and showed to participate to the innate immune defense system of sponges by directly killing pathogens and predators. However, the influence of these molecules on microorganism phagocytosis remains unexplored.

### 5.4. Association with Microorganisms

Sponges are also described as holobionts that host a stable and permanent microbial community [124]. Furthermore, it has been pointed out that sponges were able to discriminate between bacteria, which are phagocytosed for nutrition, and bacteria, which resist to the digestion. The associated bacteria are stably integrated into the sponge mesohyl or in specialized cells, named bacteriocytes [125]. So far, 47 phyla of bacteria have been described, living in close association with sponges [126]. Some bacteria showed to be to be specific to sponge holobionts, as illustrated with bacteria of the Poribacteria phylum [127], which revealed to be able to express membrane proteins with eukaryotic-like protein domains that might be recognized by sponge cells [128,129]. However, although molecular cross-talks between sponge and bacteria have been demonstrated for bacterial homoserine lactones [130], the communication between microorganisms and their sponge host remains obscure.

The role of lectins in the communication between the sponge host and its associated microorganisms is poorly documented. The *H. panicea* lectin is the only sponge lectin, which proved to be able to stimulate the *in vitro* proliferation of the associated strain *Pseudomonas insolita*, whereas no proliferative activity was shown for the other commensally isolated bacteria [50]. Authors also demonstrated that this growth stimulation was prevented either by addition of a polysaccharide-containing fraction from this bacterium into the culture medium or by heating, proving the specific activity of this lectin. However, none study was reported on a sponge lectin symbiotic activity, although the role of carbohydrates was previously investigated and recognized for playing a crucial role in the colonization and settlement of symbionts within the host.

In some associations, the symbionts recognize sugars from the host as shown with the vertebrate intestine symbiont *Bacteriodes thetaiotaomicron*, which proved to initiate the colonization of intestine in response to fucosylated glycan production by the epithelium cells [131,132]. Another study performed with *Xenorhabdus nematophila* bacterial symbionts revealed that they adhere to clusters of spherical bodies inside the intestine of the parasite nematode *Steinernema carpocapsae*, within a mucus-like material composed of *N*-acetyl-glucosamine or *N*-acetyl-galactosamine [133]. Similarly, during *Vibrio fischeri* recruitment by the *Euprymna* squid, the animal produces a mucus-like material, in which the symbiont is accumulated [134]. The symbiont showed to bind the *N*-acetyl-d-glucosamine or *N*-acetyl-d-galactoside-containing sugars within the mucus [135]. On the contrary, in other associations, the symbiont revealed to be the source of the lectin-bound carbohydrate. For example, within the association between the *Symbiodinium* sp. dinoflagellate and the octocoral *Sinularia lochmodes*, a galactose binding lectin (SLL-2) was detected on the surface of symbiotic dinoflagellate cells. The authors deduced from these data that lectins could be the mediators of symbiotic associations [136]. Similarly, the marine nematode *Laxus oneistus* revealed to express a lectin in its mucus, which showed to possess a specific carbohydrate domain involved in the recognition and recruitment of sulfur-oxidizing bacterial symbionts [137]. This nematode lectin showed a similar sequence homology with the human dendritic cell-specific immunoreceptor, suggesting a tiny frontier between symbiotic and parasitic interactions [138]. Regarding the plant lectins, it has been demonstrated that they could mediate the first steps of the symbiont settlement within the legume-rhizobium symbiosis [139]. Specific recognitions between host lectins and symbiont polysaccharides were recognized as promoting the attachment of bacteria onto the roots, enhancing the secretion of the Nod factor and triggering the bacterial infection [140,141].

These few examples highlight the potential role of lectins in the communication between the host and its associated microorganisms and reinforce the usefulness of a deeper exploration of sponge lectins since carbohydrate recognition demonstrated to play a crucial role in their symbiosis.

In conclusion, sponge lectins exhibited various activities related to the physiology of the sponge. Their carbohydrate-binding specificity confers them the ability to be involved in recognition phenomenon, especially in self and non-self recognition and to fight against pathogens. Sponges use their ability to form complexes or gel-like scaffolds as a platform to build a more complex structure in a soft/fluid environment such as the mesohyl. A future challenge would be to investigate their roles in the association between sponge and associated microorganisms.

## 6. Biotechnological Potential

Although Porifera lectins were formerly purified in order to investigate their physiological roles, their agglutinating activity on human erythrocytes highlighted their biotechnological potencies. The first sponge lectin, which demonstrated the capacity to stimulate the cell division of human lymphocytes, was isolated from the marine sponge *G. cydonium* [68]. Thus far, 39 sponge lectins have been isolated, which have revealed a huge diversity of activities including modulation of mammalian immunity, fight against human and mammalian pathogens, cancer detection and destruction, as well as modulation of neuronal activity (Table 3).

### 6.1. Activities on Mammalian Immune Cells

Some sponge lectins exhibited *in vitro* mitogenic activities on human or murine immune cells. Although such activities have not been identified within sponges, this complex specific activity of lectins on vertebrate immune cells has to be noted.

The *A. corrugata* lectin-I strongly enhanced the mitogenic activity of human mononuclear cells at concentrations of 32 to 50 μg/mL with a maximum activity at 32 μg/mL [63].

In the same manner, the *A. papillata* I [48], the *C. alloclada* [84], the *C. australiensis* [61], the *C. crambe* [78], the *C. nucula* [85], *the D. anchorata* [86], the *G. cydonium* [68] and the *H. cratera* lectins [54] showed stimulation of the human lymphocyte cell division. The *C. crambe* lectin showed to stimulate the incorporation of [^3^H]dThd in human lymphocytes with a dose-dependent response from 1 μg/mL of lectin, reaching 15-fold of the control sample incorporation rate [78]. *C. nucula* lectin strongly enhanced the mitogenic activity of H9 T_4_-lymphocyte cell line (0.3 μg/mL of lectin increased cell proliferation by a factor of 2), whereas *H. cratera* lectin exhibited a weak mitogenic effect on phytohaemagglutinin-pretreated T-lymphocytes (23% of increase of cell proliferation at 15 μg/mL of lectin) [54,85].

Similar mitogenic effects were observed for CauL murine immune cell cultures, which exibited a maximum stimulation of 53% at 0.78 μg/mL of lectin on BALB/c splenocytes, compared to control culture [61]. Both the *C. nucula* and the *P. semitubulosa* lectins showed a high mitogenic activity on B and T lymphocytes, increasing the [^3^H]Tdhd incorporation into the cells by 178% and a factor of 10, respectively, at 0.3 μg/mL of lectin [58,85]. Moreover, murine macrophages showed to be able to internalize the PsL into their cells by endocytosis.

Some sponge lectins also possess chemotaxis activity on neutrophils, as illustrated with *A. corrugata* lectin, which showed a non-dose-dependent effect from 0.4 μg/mL of lectin [53]. In contrast, the *C. varians* lectin increased the *in vivo* chemotaxis of murine neutrophils in the peritoneal cavity with a maximum stimulation of 400%, in comparison with the negative control, 4 h after CvL injection (50 μg/mL) [142].

Other lectins showed to stimulate the production of cytokines by immune cells. For example, PsL demonstrated to enhance the production of interleukin-1 and -2 in murine peritoneal macrophage and lymphocyte cultures [58].

Sponge lectins can play the function of opsonin, by activating the multiplication of immune cells, attracting neutrophil cells, stimulating the production of cytokines and triggering their endocytosis. Similarly to sponge lectins, the vertebrate galectin 3 was able in some conditions to develop the pro-inflammatory response. The superoxide production increased in neutrophil cultures in presence of recombinant human galectin 3, suggesting their activation [143]. A decrease of peritoneal inflammatory responses against pathogens was observed with galectin 3 deficient (*gal3*^−/−^) mice. *Gal3*^−/−^ mice developed less inflammation in the peritoneal cavity than in the wild type mice. Activation of inflammatory cells by thioglycollate caused lower levels of NFκB response in *gal3*^−/−^ mice. These results support the inflammatory modulating activity of the multifaceted galectin 3. The morphology of macrophages from deficient mice demonstrated to be spindle-shaped, whereas galectin 3 promoted the cell survival of peritoneal macrophages [144]. Human galectin 3 also displayed chemotactic activity on neutrophils and macrophages as described for AcL and CvL [53,142,145]. Pro-inflammatory properties were also observed for lectins from other organisms, such as the algae *Ticocarpus crinitus* and the mussel *Mytilus trossulus* lectins, which showed to stimulate the cytokine production and the lymphocyte proliferation [14,15].

Despite the lack of specialized immune cells in sponges, their lectins are able to act on vertebrate cells as immunomodulatory molecules and seem to be functionally related to a vertebrate galectin. This latter property could be used against diseases triggering a depletion of the immune system.

### 6.2. Antimicrobial Activities

Thanks to their ability to recognize specific sugar residues, some sponge lectins can bind specific carbohydrates of microbe cell walls and consequently exhibit antimicrobial activities.

The *C. varians* lectin displayed antibacterial activity against the Gram positive bacteria *Bacillus* *subtilis* and *Staphylococcus aureus*. A growth inhibition of 75% and 90% against *B. subtilis* was observed after treatment with 25 and 100 μg/mL of CvL, respectively. This antibacterial effect was also reported against the bacterium *S. aureus*, with 90% of growth inhibition at 50 μg/mL, compared to the control culture [59]. Furthermore, the *S. domuncula* lectin cDNA displayed high similarities with tachylectin 1 cDNA, also called lectin L6, from hemocytes of the horseshoe crab *Tridentatus trunculus* [41]. Investigation of the antibacterial activity of this lectin against *E. coli* and *S. aureus* only revealed a weak activity (16%) against *E. coli* at 10 μg/mL (81% of growth inhibition at 300 μg/mL). The *S. domuncula* lectin specifically binds LPS, which are the major components of Gram-negative bacteria cell walls. This antibacterial activity was already evaluated in hemocytes from the horseshoe crab *T. trunculus*, for which several antibacterial lectins were isolated and identified as tachylectins 1, 2, 3 and 4. Tachylectin 1 showed antibacterial activity against Gram-negative bacteria [40]. Tachylectin 2 exhibited specificity to bind *N*-acetylglucosamine (GlcNAc) and *N*-acetylgalactosamine (GalNAc) and was found to agglutinate several *S. aureus* strains [146,147]. Tachylectin 3 and tachylectin 4 targeted the LPS of Gram-negative bacteria, as described for Sd lectin [148,149]. This ability of sponge antibacterial lectins to target specific carbohydrates could potentially be used to develop antibiotics against resistant bacterial infections.

The galactose-binding *C. nucula* lectin displayed antiviral activities. This lectin increased the infected cell-releasing period of the HIV-I, which might be correlated with an increasing expression of the (2′-5′) oligoriboadenylate synthetase in the infected cells in the presence of the lectin [85]. The antiviral potency of lectins was already demonstrated with the cyanovirin lectin, which showed to inhibit the penetration of HIV, Ebola, influenza and herpes viruses in host cells by linking the mannose residues of the viral envelops [150]. This mechanism was also observed for *Kappaphycus alvarezii* and *Booglea coacta* red algae lectins [19,20]. By a different mechanism of action, the *C. variopedatus* and *S. vermicularis* sea worm lectins revealed to act by inhibiting the viral antigen production, preventing the cytopathic effect of viruses [22,23].

Some sponge lectins also demonstrated to be able to agglutinate the parasite *Leishmania chagasi*. The *C. varians* lectin exhibited agglutinating activity against promastigotes of *L. chagasi*, suggesting a possible application to fight against pathogenic protozoa. According to the two-fold serial dilution method, this lectin was able to agglutinate up to a titer of eight agglutinating units (AU) that correspond to agglutination of 10^6^ cells by 1 μg of *C. varians* lectin [59]. The *C. apion* lectin showed a similar activity, agglutinating up to AU titer of 4 (6.7 × 10^5^ cells for 1 μg of *C. apion* lectin). The agglutinating activity of both lectins was inhibited in presence of galactoside residues-containing carbohydrates (galactose and lactose) [60]. Similarly, lectins from plant extracts of *Ricinus communis*, *Capparis spinosa*, *Prosopis farcta* and *Tamarix nilotica* showed to agglutinate and to kill the parasite *Leishmania major* promastigote *in vitro*. Authors demonstrated that ingestion of the *R. communis* extract by the insect vector *Phlebotomus papatasi* increased the *in vivo* mortality of the parasites within the sandfly *P. papatasi* [151]. These results suggest that *L. chagasi* promastigotes have specific glycosylated receptors for these lectins on the cell membrane of the parasite. Specificities of parasite receptors have extensively been studied, especially for trypanosomatids [152,153,154,155]. These lectins may represent valuable tools for the detection and fight against the protozoan parasites. Similarly, a protective effect was reported for the lectin from the plant *Synadenium carinatum* on *Leishmania amazonensis* infection of BALB/c mice, highlighting its potency as an adjuvant in murine model of vaccination against American cutaneous leishmaniasis [156].

**Table 3:** Biological properties of sponge lectins.

Name	Species	Order (Class)	Biological Activities	Physiological Roles	References
**Sponge galectins**					
CchG 1	*Cinachyrella sp.*	Spirophorida (D)	rabbit erythrocyte agglutination modulatory activity of human glutamate receptors	nd	[65]
CchG 2	*Cinachyrella sp.*	Spirophorida (D)	rabbit erythrocyte agglutination	nd	[65]
GCG	*Geodia cydonium*	Astrophorida (D)	modulatory activity of human glutamate receptors increase of the growth rate of L5178y mouse lymphoma cells mitotic activity on human lymphocytes	cell interaction	[68,69,70,71,72]
HoL-30	*Halichondria okadai*	Halichondrida (D)	rabbit and human erythrocyte agglutination	nd	[62]
Sd galectin 1	*Suberites domuncula*	Hadromerida (D)	nd	canal system formation in primmorphs	[51]
Sd galectin 2	*Suberites domuncula*	Hadromerida (D)	nd	biomineralization/spiculogenesis	[67]
**Sponge C-type lectins**					
AaL	*Aplysina archeri*	Verongida (D)	hamster, rabbit, bovine and human erythrocyte agglutination	nd	[77]
AlL	*Aplysina lacunosa*	Verongida (D)	hamster, rabbit, bovine and human erythrocyte agglutination	nd	[77]
AvL	*Aphrocallistes vastus*	Hexactinosida (H)	nd	cell interaction	[56]
CvL	*Cliona varians*	Hadromerida (D)	human erythrocte agglutination antibacterial activity against *B. subtilis* and *S. aureus* no activity against *E. coli* and *P. aeruginosa* *Leishmania chagasi* agglutination chemotactic on mouse neutrophils *in vivo* cytotoxic activity against K562 and Jurkat cells no cytotoxicity on human erythrocytes and blood cells	nd	[59,142,157]
Lb MBL	*Lubomirskia baicalensis*	Haplosclerida (D)	nd	biomineralization/spiculogenesis	[52]
PsL	*Pellina semitubulosa*	Halichondrida (D)	sheep, rabbit and human erythrocyte agglutination strong mitogenic effect on spleen lymphocytes of mice interleukin-1 release from mouse peritoneal macrophages nterleukin-2 production by murine lymphocyte cultures	nd	[58]
**Sponge tachylectin-like lectins**					
Ef lectin	*Ephydatia fluviatilis*	Haplosclerida (D)	putative antibacterial activity	host defense	[42]
Sd lectin	*Suberites domuncula*	Hadromerida (D)	antibacterial activity against *E. coli* and *S. aureus*	host defense	[41]
**Sponge F-type lectin**					
CcL	*Crambe crambe*	Poecilosclerida (D)	sheep and human erythrocyte agglutination mitotic activity on human lymphocytes	nd	[78]
**Unclassified sponge lectins**					
AcL I	*Axinella corrugata*	Halichondrida (D)	goat, dog and rabbit erythrocyte agglutination chemotaxis activity on rat neutrophils mitotic effect toward human mononuclear cells cytotoxic effect against *Artemia salina*	host defense	[53,63]
AcL II	*Axinella corrugata*	Halichondrida (D)	rabbit erythrocyte agglutination	nd	[63]
ApaL I	*Aaptos papillata*	Hadromerida (D)	nd	nd	[48]
ApaL II	*Aaptos papillata*	Hadromerida (D)	nd	nd	[48]
ApaL III	*Aaptos papillata*	Hadromerida (D)	nd	nd	[48]
ApL I	*Axinella polypoides*	Halichondrida (D)	mitogenic activation on human lymphocytes	spongin production	[64,87]
ApL II	*Axinella polypoides*	Halichondrida (D)	nd	spongin production	[64,87]
ApL III	*Axinella polypoides*	Halichondrida (D)	nd	nd	[45,79]
ApL IV	*Axinella polypoides*	Halichondrida (D)	nd	nd	[45,79]
ApL V	*Axinella polypoides*	Halichondrida (D)	nd	nd	[45,79]
CaL	*Cinachyrella apion*	Spirophorida (D)	human erythrocte agglutination *Leishmania chagasi* agglutination antiproliferative activity against HeLa, PC3 and 3T3 cells no cytotoxicity on human erythrocytes and blood cells	host defense	[60,158]
CalL	*Cinachyrella alloclada*	Spirophorida (D)	human erythrocyte aggulitination	nd	[84]
CauL	*Craniella australiensis*	Spirophorida (D)	mitogenic activity on human lymphocytes mouse, sheep, rabbit and human erythrocte agglutination mitogenic activity on BALB/c splenocytes	host defense	[61]
CnL	*Chondrilla nucula*	Chondrosida (D)	mitotic activity on mouse and human lymphocytes increase the activity of the (2′-5′) oligoadenylate modulation of HIV-release period by infected cells	host defense	[85]
CtL	*Cinachyrella tenuiviolacea*	Spiroporida (D)	human erythrocte agglutination	nd	[46]
DaL	*Desmapsamma anchorata*	nd	human erythrocte agglutination mitogenic activity on human lymphocytes	nd	[86]
Halilectin 1 (H-1)	*Haliclona caerulea*	Haplosclerida (D)	rabbit erythrocyte agglutination cytotoxic effect on *Artemia nauplii*	host defense	[49]
Halilectin 2 (H-2)	*Haliclona caerulea*	Haplosclerida (D)	rabbit erythrocyte agglutination cytotoxic effect on *Artemia nauplii*	host defense	[49]
Halilectin 3 (H-3)	*Haliclona caerulea*	Haplosclerida (D)	human and rabbit erythrocyte agglutination	nd	[83]
HcL	*Haliclona cratera*	Haplosclerida (D)	sheep and human erythrocyte agglutination cytotoxic activity against HeLa and FemX cells weak mitogenic effect on human T lymphocytes	nd	[54]
HL	*Haliclona sp.*	Haplosclerida (D)	human erythrocte agglutination	nd	[46]
HoL-1	*Halichondria okadai*	Halichondrida (D)	human erythrocte agglutination	nd	[80]
HoL-2	*Halichondria okadai*	Halichondrida (D)	human erythrocte agglutination	nd	[80]

nd: not determined; D: Demospongiae; H: Hexactinellidae.

### 6.3. Cytotoxic and Anticancer Activities

Some sponge lectins also displayed cytotoxic effects towards cancer cell lines. The cytotoxic activity of the *H. cratera* lectin (*Haliclona cratera*) was studied in HeLa cells (laboratory-cultured strain of a human cervical cancer) and FemX cells (laboratory-cultured strain of a human melanoma) using the MTT (3-(4,5-dimethylthiazol-2-yl)-2,5-diphenyltetrazolium bromide) assay. According to the authors, the highest lectin concentrations, which led to a decrease of 50% (IC_50_) in cell survival, was 9 μg/mL and 11 μg/mL for HeLa and FemX cells, respectively [54]. The *C. varians* lectin also exhibited cytotoxic effects against the K562 erythroleukemia cell line (derived from a chronic myeloid leukemia) and the Jurkat cell line (a human T-cell leukemia cell line) (American Type Culture Collection (ATCC), Rockeville, MD, USA). The proliferation of the K562 cell line was dose-dependently inhibited by CvL (IC_50_ = 70 μg/mL); the cell viability reached a maximum decrease (25%) at 80 μg/mL of CvL. Jurkat cells were less sensitive to this lectin with an IC_50_ of 100 μg/mL. Conversely, no cytotoxic effect was detected against normal human lymphocytes [157]. The *C. apion* lectin was tested against HeLa, PC3 (human prostate adenocarcinoma) and 3T3 (immortalized mouse fibroblast line) cell lines. CaL caused a dose-dependent inhibition of cell proliferation in HeLa and PC3 cells. The IC_50_ for HeLa cells was obtained at a concentration of 10 μg/mL of CaL. Toxicity of CaL was demonstrated against 3T3 cells, without a significant effect on cell proliferation. The MTT assay did not reveal any cytotoxicity of CaL either on erythrocytes and peripherical blood cells or on solid tumors [158].

The mechanism underlying the toxicity of sponge lectins on cancer cell lines was explored for both CaL and CvL. CaL showed to increase the expression of tumor necrosis factor α receptor-1 (TNFR-1) and p21 protein and to decrease the expression of nuclear factor κB p65 (NFκB) subunit and pRb protein in K562 cell line. This regulation suggests an induction of apoptosis and the cell cycle arrest. The lysosomal protease cathepsin B showed to play a central role in CvL-mediated apoptosis. CvL killed K652 cells mainly via a caspase-dependent mechanism, potentially involving the death receptor pathway [157]. The mechanism used by CaL to induce apoptosis of HeLa and 3T3 cells was mediated by caspase-independent and -dependent activities. The expression of Bax and NFκB proteins increased in the presence of CaL lectin until 18 h of treatment before decreasing, but this overexpression was not observed for BcL-2 protein. CaL induces apoptosis via mitochondrial membrane permeabilization, promoting the release of cytochrome C, AIF (apoptosis-inducing factor) and/or endonuclease G [158].

In conclusion, sponge lectins have demonstrated to act on cancer cell lines through inhibiting cell proliferation and promoting apoptosis by different pathways. The specific binding of these lectins on non-solid tumors could be used to discriminate cancerous cells from normal cells, as membrane carbohydrates are different in tumor cells. These carbohydrates can be specifically recognized by lectins. Similarly to CaL, the plant *Viscum album* Mistletoe lectin I demonstrated to activate the caspase independently to the activation of the cell death receptor, through the release of cytochrome C into the cytosol of leukemic T- and B-cell lines [159]. The jack bean concanavalin A revealed to differently activate the apoptosis of ovarian SKOV3 cells and melanoma A375, triggering the activation of the Foxola-Bim signaling pathways or enhancing the release of cytochrome C from mitochondria, respectively. These results suggest that the same lectin, in this case concanavalin A, can differently act according to the type of cells [9,160]. Several lectins, including the plant *Viscum album* Mistletoe, the hexacoral *Gerardia savaglia* GSL and the jack bean Concanavalin A lectins, have been used to differentiate malignant tumors from benign tumors and the degree of glycosylation associated with metastasis [159,160,161].

### 6.4. Neuromodulatory Activity

By searching for molecules with neuromodulatory activities in sponge extracts [65], the authors set-up a bioassay in order to detect active molecules on mammalian ionotropic glutamate receptors, expressed in HEK293-T/17 cells. Both *Cinachyrella* sp. galectins 1 and 2 were subsequently isolated from a *Cinachyrella* sp. marine sponge. A 5-min application of CchGs (10 μg/mL) slowed down the time of desensitization of these currents and increased the amplitude of steady-state currents. The recombinant *Cinachyrella* sp. galectin a (1 μg/mL) slowed down in the same way glutamate-evoked currents from homomeric GluK2 kainate receptors expressed in HEK293-T/17 cells [65]. This inhibition was lost in the presence of lactose. Despite the lack of a nervous system in sponges, neural cell-specific proteins have already been found in these animals, such as a metabotropic glutamate receptor-like, potentially regulating membrane ionic canal activity in the demosponges *S. domuncula* and *G. cydonium* [162]. These data show that sponge lectins could act on the nervous system of higher animals. This neuromodulatory effect on vertebrate brains was already observed with plant lectins. The plant ConBr lectin, isolated from *Canavalia brasiliensis*, showed a nervous antidepressant-like activity. This lectin caused a potentiation of the fluoxetine action, a selective serotonin reuptake inhibitor, leading to a decrease of the mouse immobility time during forced swimming tests [163]. Furthermore, rats treated with the *Canavalia ensiformis* concanavalin A dose-dependently demonstrated a velocity decrease of the cortical spreading depression propagation and an increase of the amplitude and the duration of the cortical spreading depression potential [164].

In conclusion, the sponge lectins display diverse activities, but all their biotechnological and medical potencies have not totally been explored, as illustrated by the intriguing neuromodulating and anticancer activities of the *Cinachyrella* sp. and the *C. apion* lectins, respectively [65,158].

## 7. Conclusions

In this review, we report on methods used to investigate sponge lectins and to classify them according to their biochemical characteristics. We showed that the purification of sponge lectins was usually performed by using affinity chromatography followed by gel filtration, even if some original protocols were developed. According to their binding activities, Porifera lectins have been classified into lectin groups, including galectins, C-type, F-type and tachylectin-like lectins. Because of a lack of data, unclassified sponge lectins are presented according to their characteristics as intrachain disulfide bridge-containing, mucin-binding and *N*-acetyl-galactosamine/*N*-acetyl-glucosamine-binding lectins.

Currently, the development of sponge cDNA libraries allows the direct access to lectin gene sequences and their production in heterologous systems in order to evaluate their bioactivities.

Sponge lectins displayed a broad range of bioactivities including mitogenic, antimicrobial, antitumor and neuromodulatory activities. Some of these activities were attributed to physiological functions within the sponge, such as cell adhesion and differentiation, spiculogenesis and host defense. Therefore, in regard to their different interesting bioactivities, sponge lectins also constitute a source of biotechnologically and medically interesting molecules. Their carbohydrate-binding properties and their biological activities allow expecting their development either as additional drugs in some diseases or as specific biomarkers.

A particular interest was reported on demosponge lectins because their biomass is more compatible with lectin purification. Only one hexactinellid lectin has been studied so far, but none lectin was isolated from the class of Calcarea or Homoscleromorpha. Taking into account of the diversity and originality of demosponge lectin structures and their bioactivities, it would be interesting to increase the efforts in purification of lectins from the other Porifera classes. The discovery of new sponge lectins should lead to an in-depth investigation of their physiological functions, especially the symbiont recruitment, in order to consider their potential as molecules/tools for new biotechnological and medical applications.

## References

[1] Boyd W.C., Sharpleigh E. (1954). Specific precipitation activity of plant agglutinins (lectins). Science.

[2] Gabius H.J. (1997). Animal lectin. J. Biochem..

[3] Rüdiger H., Gabius H.J. (2001). Plant lectins: Occurrence, biochemistry, functions and applications. Glycoconj. J..

[4] Nascimento K.S., Cunha A.I., Nascimento K.S., Cavada B.S., Azevedo A.M., Aires-Barros M.R. (2012). An overview of lectins purification strategies. J. Mol. Recognit..

[5] Sharon N. (1987). Bacterial lectins, cell-cell recognition and infectious disease. FEBS.

[6] Lakhtin V., Lakhtin M., Alyoshkin V. (2011). Lectins of living organisms. The overview. Anaerobe.

[7] Sumner J.B., Howel S.F. (1936). Identification of hemagglutinin of jack bean with Concanavalin A. J. Bacteriol..

[8] Hamblin J., Kent S.P. (1973). Possible role of phytohaemagglutinin in *Phaseolus vulgaris* L.. Nat. New Biol..

[9] Jiang Q.L., Zhang S., Tian M., Zhang S.Y., Xie T., Chen D.Y., Chen Y.J., He J., Liu J., Ouyang L. (2015). Plant lectins, from ancient sugar-binding proteins to emerging anti-cancer drugs in apoptosis and autophagy. Cell Prolif..

[10] Ang A.S., Cheung R.C., Dan X., Chan Y.S., Pan W., Ng T.B. (2014). Purification and characterization of a glucosamine-binding antifungal lectin from *Phaseolus vulgaris* cv. Chinese pinto beans with antiproliferative activity towards nasopharyngeal carcinoma cells. Appl. Biochem. Biotechnol..

[11] Ashraf M.T., Khan R.H. (2003). Mitogenic lectins. Med. Sci. Monit. Int. Med. J. Exp. Clin. Res..

[12] Valadez-Vega C., Guzmán-Partida A.M., Soto-Cordova F.J., Alvarez-Manilla G., Zúñiga-Pérez C., Morales-González J.A., Madrigal-Santillán E., Villagómez-Ibarra J.R., Gutiérrez-Salinas J., Becerril-Flores M.A. (2011). Purification, biochemical characterization, and bioactive properties of a lectin purified from the seeds of white tepary bean (*Phaseolus acutifolius* variety latifolius). Molecules.

[13] Chernikov O.V., Molchanova V.I., Chikalovets I.V., Kondrashina A.S., Li W., Lukyanov P.A. (2013). Lectins of marine hydrobionts. Biochem. Mosc..

[14] Molchanova V.I., Chernikov O.V., Chikalovets I.V., Lukyanov P.A. (2010). Purification and partial characterization of the lectin from the marine red alga *Tichocarpus crinitus* (Gmelin) Rupr. (Rhodophyta). Bot. Mar..

[15] Chikalovets I.V., Kondrashina A.S., Chernikov O.V., Molchanova V.I., Lukyanov P.A. (2013). Isolation and general characteristics of lectin from the mussel *Mytilus trossulus*. Chem. Nat. Comp..

[16] O’Keefe B.R., Giomarelli B., Barnard D.L., Shenoy S.R., Chan P.K., McMahon J.B., Palmer K.E., Barnett B.W., Meyerholz D.K., Wohlford-Lenane C.L. (2010). Broad-spectrum *in vitro* activity and *in vivo* efficacy of the antiviral protein griffithsin against emerging viruses of the family Coronaviridae. J. Virol..

[17] Meuleman P., Albecka A., Belouzard S., Vercauteren K., Wychowski C., Leroux-Roels G., Verhoye L., Palmer K.E., Dubuisson J. (2011). Griffithsin has antiviral activity against hepatitis C virus. Antimicrob. Agents Chemother..

[18] Ishag H.Z., Li C., Huang L., Sun M.X., Wang F., Ni B., Malik T., Chen P.Y., Mao X. (2013). Griffithsin inhibits Japanese encephalitis virus infection *in vitro* and *in vivo*. Arch. Virol..

[19] Sato Y., Morimoto K., Hirayama M., Hori K. (2011). High mannose-specific lectin (KAA-2) from the red alga *Kappaphycus alvarezii* potently inhibits influenza virus infection in a strain-independent manner. Biochem. Biophys. Res. Commun..

[20] Sato Y., Hirayama M., Morimoto K., Yamamoto N., Okuyama S., Hori K. (2012). *Boodlea coacta* is a potent entry inhibitor of HIV-1 and influenza viruses. J. Biol. Chem..

[21] Li W., Wang J.H., Dong-Yun O.Y., Molchanova V.I., Chikalovets I.V., Chernikov O.V. (2006). Anti-human immunodeficiency virus type 1 (HIV-1) activity of lectins from ascidian *Didemnum ternatanum*. Glycobiology.

[22] Wang J.H., Kong J., Li W., Molchanova V.I., Chikalovets I.V., Belogortseva N., Lukyanov P.A., Zheng Y.T. (2006). A beta-galactose-specific lectin isolated from the marine worm *Chaetopterus variopedatus* possesses anti-HIV-1 activity. Comp. Biochem. Physiol. C Toxicol. Pharmacol..

[23] Molchanova V.I., Chikalovets I.V., Chernikov O.V., Belogortseva N., Li W., Wang J.H., Yang D.Y., Zheng Y.T., Lukyanov P. (2007). A new lectin from the sea worm *Serpula vermicularis*: Isolation, characterization and anti-HIV activity. Comp. Biochem. Physiol. C Toxicol. Pharmacol..

[24] Kovalchuk S.N., Chikalovets I.V., Chernikov O.V., Molchanova V.I., Li W., Rasskazov V.A., Lukyanov P.A. (2013). cDNA cloning and structural characterization of a lectin from the mussel *Crenomytilus grayanus* with a unique amino acid sequence and antibacterial activity. Fish Shellfish Immunol..

[25] Van Soest R.W., Boury-Esnault N., Vacelet J., Dohrmann M., Erpenbeck D., de Voogd N.J., Santodomingo N., Vanhoorne B., Kelly M., Hooper J.N. (2012). Global diversity of sponges (Porifera). PLoS ONE.

[26] Kim S.K., Dewapriya P. (2012). Bioactive compounds from marine sponges and their symbiotic microbes: A potential source of nutraceuticals. Adv. Food Nutr. Res..

[27] Roué M., Quévrain E., Domart-Coulon I., Bourguet-Kondracki M.L. (2012). Assessing calcareous sponges and their associated bacteria for the discovery of new bioactive natural products. Nat. Prod. Rep..

[28] Privat de Garilhe M., de Rudder J. (1964). Effet de deux nucleosides de l’arabinose sur la multiplication des virus de l’herpes et de la vaccine en culture cellulaire. C. R. Acad. Sci..

[29] Momparler R.L. (1967). A model for chemotherapy of acute leukemia with 1-β-d-arabinofuranosylcytosine. Cancer Res..

[30] Bergmann W., Feeney R.J. (1951). Contributions to the study of marine products. XXXII. The nucleosides of sponges. I. J. Org. Chem..

[31] Hirata Y., Uemura D. (1986). Halichondrins-antitumor polyether macrolides from a marine sponge. Pure Appl. Chem..

[32] Traynor K. (2011). Eribulin approved for advanced breast cancer. Am. J. Health Syst. Pharm..

[33] Prokop O., Uhlenbruck G., Kohler W. (1968). A new source of antibody-like substances having anti-blood group specificity. A discussion on the specificity of *Helix* agglutinins. Vox Sang..

[34] Baldo B.A., Uhlenbruck G. (1975). Tridacnin, a potent anti-galactan precipitin from the hemolymph of *Tridacna maxima* (Röding). Adv. Exp. Med. Biol..

[35] Müller W.E.G., Kurelec B., Zahn R.K., Müller I.M., Vaith P., Uhlenbruck G. (1979). Aggregation of sponge cells. Function of a lectin in its homologous biological system. J. Biol. Chem..

[36] Gomes Filho S.M., Cardoso J.D., Anaya K., Silva do Nascimento E., de Lacerda J.T., Mioso R., Santi Gadelha T., de Almeida Gadelha C.A. (2015). Marine sponge lectins: Actual status on properties and biological activities. Molecules.

[37] Hirabayashi J., Kasai K. (1993). The family of metazoan metal-independent beta-galactoside-binding lectins: Structure, function and molecular evolution. Glycobiology.

[38] Müller W.E.G., Blumbach B., Wagner-Hülsmann C., Lessel U. (1997). Galectins in the phylogenetically oldest metazoa, the sponge (Porifera). Trends Glycosci. Glycotechnol..

[39] Vasta G.R., Ahmed H., Bianchet M.A., Fernández-Robledo J.A., Amzel L.M. (2012). Diversity in recognition of glycans by F-type lectins and galectins: Molecular, structural, and biophysical aspects. Ann. N. Y. Acad. Sci..

[40] Saito T., Kawabata S., Hirata M., Iwanaga S. (1995). A novel type of limulus lectin-L6. Purification, primary structure, and antibacterial activity. J. Biol. Chem..

[41] Schröder H.C., Ushijima H., Krasko A., Gamulin V., Thakur N.L., Diehl-Seifert B., Müller I.M., Müller W.E.G. (2003). Emergence and disappearance of an immune molecule, an antimicrobial lectin, in basal metazoa. A tachylectin-related protein in the sponge *Suberites domuncula*. J. Biol. Chem..

[42] Funayama N., Nakatsukasa M., Kuraku S., Takechi K., Dohi M., Iwabe N., Miyata T., Agata K. (2005). Isolation of Ef silicatein and Ef lectin as molecular markers for sclerocytes and cells involved in innate immunity in the freshwater sponge *Ephydatia fluviatilis*. Zool. Sci..

[43] Dodd R.Y., MacLennan A.P., Alyoshkin V. (1968). Haemagglutinins from marine sponges. Vox Sang..

[44] Phillips S.G., Bretting H., Kabat E.A. (1976). A galactose-inhibitable mitogen for human lymphocytes from the sponge *Axinella polypoides*. J. Immunol..

[45] Bretting H., Donadey C., Vacelet J., Jacobs G. (1981). Investigations on the occurrence of lectins in marine sponges with special regard to some species of the familly axinellidae. Comp. Biochem. Physiol. B.

[46] Mebs D., Weiler I., Heinke H.F. (1985). Bioactive proteins from marine sponges: Screening of sponge extracts for hemagglutinating, hemolytic, ichthyotoxic and lethal properties and isolation and characterization of hemagglutinins. Toxicon.

[47] Kljajic Z. (1986). Faculty of Chemistry. Ph.D. Thesis.

[48] Bretting H., Kabat E.A., Liao J., Pereira M.E. (1976). Purification and characterization of the agglutinins from the sponge *Aaptos papillata* and a study of their combining sites. Biochemistry.

[49] Carneiro R.F., de Melo A.A., Nascimento F.E., Simplicio C.A., Nascimento K.S., Rocha B.A., Saker-Sampaio S., Moura Rda M., Mota S.S., Cavada B.S. (2013). Halilectin 1 (H-1) and Halilectin 2 (H-2): Two new lectins isolated from the marine sponge *Haliclona caerulea*. Int. J. Biochem. Cell Biol..

[50] Müller W.E.G., Zahn R.K., Kurelec B., Lucu C., Müller I.M., Uhlenbruck G. (1981). Lectin, a possible basis for symbiosis between bacteria and sponges. J. Bacteriol..

[51] Wiens M., Mangoni A., D’Esposito M., Fattorusso E., Korchagina N., Schröder H.C., Grebenjuk V.A., Krasko A., Batel R., Müller I.M. (2003). The molecular basis for the evolution of the metazoan bodyplan: Extracellular matrix-mediated morphogenesis in marine demosponges. J. Mol. Evol..

[52] Wiens M., Belikov S.I., Kaluzhnaya O.V., Krasko A., Schröder H.C., Perovic-Ottstadt S., Müller W.E.G. (2006). Molecular control of serial module formation along the apical-basal axis in the sponge *Lubomirskia baicalensis*: Silicateins, mannose-binding lectin and mago nashi. Dev. Genes Evol..

[53] Dresch R.R., Zanetti G.D., Lerner C.B., Mothes B., Trindade V.M., Vozári-Hampe M.M., Henriques A.T. (2008). ACL-I, a lectin from the marine sponge *Axinella corrugata*: Isolation, characterization and chemotactic activity. Comp. Biochem. Physiol. C Toxicol. Pharmacol..

[54] Pajic I., Kljajic Z., Dogovic N., Sladic D., Juranic Z., Gasic M.J. (2002). A novel lectin from the sponge *Haliclona cratera*: Isolation, characterization and biological activity. Comp. Biochem. Physiol. C Toxicol. Pharmacol..

[55] Hanisch F.G., Baldus S.E., Kümmel T.A. (1996). Forssman disaccharide is the specific ligand of a galectin from the sponge *Geodia cydonium* but does not mediate its binding to nuclear protein np56. Glycobiology.

[56] Gundacker D., Leys S.P., Schröder H.C., Müller I.M., Müller W.E.G. (2001). Isolation and cloning of a C-type lectin from the hexactinellid sponge *Aphrocallistes vastus*: A putative aggregation factor. Glycobiology.

[57] Wagner-Hülsmann C., Bachinski N., Diehl-Seifert B., Blumbach B., Steffen R., Pancer Z., Müller W.E.G. (1996). A galectin links the aggregation factor to cells in the sponge (*Geodia cydonium*) system. Glycobiology.

[58] Engel M., Bachmann M., Schröder H.C., Rinkevich B., Kljajic Z., Uhlenbruck G., Müller W.E.G. (1992). A novel galactose- and arabinose-specific lectin from the sponge *Pellina semitubulosa*: Isolation, characterization and immunobiological properties. Biochimie.

[59] Moura R.M., Queiroz A.F., Fook J.M., Dias A.S., Monteiro N.K., Ribeiro J.K., Moura G.E., Macedo L.L., Santos E.A., Sales M.P. (2006). CvL, a lectin from the marine sponge *Cliona varians*: Isolation, characterization and its effects on pathogenic bacteria and *Leishmania* promastigotes. Comp. Biochem. Physiol. A Mol. Integr. Physiol..

[60] Medeiros D.S., Medeiros T.L., Ribeiro J.K., Monteiro N.K., Migliolo L., Vasconcelos I.M., Uchoa A.F., Oliveira A.S., de Sales M.P., Santos E.A. (2010). A lactose specific lectin from the sponge *Cinachyrella apion*: Purification, characterization, *N*-terminal sequences alignment and agglutinating activity on *Leishmania promastigotes*. Comp. Biochem. Physiol. B Biochem. Mol. Biol..

[61] Xiong C., Li W., Liu H., Zhang W., Dou J., Bai X., Du Y., Ma X. (2006). A normal mucin-binding lectin from the sponge *Craniella australiensis*. Comp. Biochem. Physiol. C Toxicol. Pharmacol..

[62] Kawsar S.M., Fujii Y., Matsumoto R., Ichikawa T., Tateno H., Hirabayashi J., Yasumitsu H., Dogasaki C., Hosono M., Nitta K. (2008). Isolation, purification, characterization and glycan-binding profile of a d-galactoside specific lectin from the marine sponge, *Halichondria okadai*. Physiol. B Biochem. Mol. Biol..

[63] Dresch R.R., Lerner C.B., Mothes B., Trindade V.M., Henriques A.T., Vozári-Hampe M.M. (2012). Biological activities of ACL-I and physicochemical properties of ACL-II, lectins isolated from the marine sponge *Axinella corrugata*. Comp. Biochem. Physiol. B Biochem. Mol. Biol..

[64] Buck F., Schulze C., Breloer M., Strupat K., Bretting H. (1998). Amino acid sequence of the d-galactose binding lectin II from the sponge *Axinella polypoides* (Schmidt) and identification of the carbohydrate binding site in lectin II and related lectin I. Comp. Biochem. Physiol. B Biochem. Mol. Biol..

[65] Ueda T., Nakamura Y., Smith C.M., Copits B.A., Inoue A., Ojima T., Matsunaga S., Sakai R., Swanson G.T. (2013). Isolation of novel prototype galectins from the marine ball sponge *Cinachyrella* sp. guided by their modulatory activity on mammalian glutamate-gated ion channels. Glycobiology.

[66] Pfeifer K., Haasemann M., Gamulin V., Bretting H., Fahrenholz F., Müller W.E.G. (1993). S-type lectins occur also in invertebrates: High conservation of the carbohydrate recognition domain in the lectin genes from the marine sponge *Geodia cydonium*. Glycobiology.

[67] Schröder H.C., Boreiko A., Korzhev M., Tahir M.N., Tremel W., Eckert C., Ushijima H., Müller I.M., Müller W.E.G. (2006). Co-expression and functional interaction of silicatein with galectin: Matrix-guided formation of siliceous spicules in the marine demosponge *Suberites domuncula*. J. Biol. Chem..

[68] Bretting H., Phillips S.G., Klumpart H.J., Kabat E.A. (1981). A mitogenic lactose-binding lectin from the sponge *Geodia cydonium*. J. Immunol..

[69] Diehl-Seifert B., Zahn R.K., Uhlenbruck G., Maidhof A., Müller W.E.G. (1985). Control of L5178y cell growth by the galactose-specific lectin from *Geodia cydonium*. Basic Appl. Histochem..

[70] Diehl-Seifert B., Uhlenbruck G., Geisert M., Zahn R.K., Müller W.E.G. (1985). Physicochemical and functional characterization of the polymerization process of the *Geodia cydonium* lectin. Eur. J. Biochem..

[71] Müller W.E.G., Conrad J., Schröder C., Zahn R.K., Kurelec B., Dreesbach K., Uhlenbruck G. (1983). Characterization of the trimeric, self-recognizing *Geodia cydonium* lectin I. Eur. J. Biochem..

[72] Stalz H., Roth U., Schleuder D., Macht M., Haebel S., Strupat K., Peter-Katalinic J., Hanisch F.G. (2006). The *Geodia cydonium* galectin exhibits prototype and chimera-type characteristics and a unique sequence polymorphism within its carbohydrate recognition domain. Glycobiology.

[73] Ohyama Y., Hirabayashi J., Oda Y., Ohno S., Kawasaki H., Suzuki K., Kaisai K. (1986). Nucleotide sequence of chick 14K beta-galactoside-binding lectin mRNA. Biochem. Biophys. Res. Commun..

[74] Rothe B., Roggentin P., Frank R., Blocker H., Schauer R. (1989). Cloning, sequencing and expression of a sialidase gene from *Clostridium sordellii* G12. J. Gen. Microbiol..

[75] Wang X., Schloßmacher U., Wiens M., Batel R., Schröder H.C., Müller W.E.G. (2012). Silicateins, silicatein interactors and cellular interplay in sponge skeletogenesis: Formation of glass fiber-like spicules. FEBS J..

[76] Freymann D.M., Nakamura Y., Focia P.J., Sakai R., Swanson G.T. (2012). Structure of a tetrameric galectin from *Cinachyrella* sp. (ball sponge). Acta Crystallogr. D Biol. Crystallogr..

[77] Miarons P.B., Fresno M. (2000). Lectins from tropical sponges. Purification and characterization of lectins from genus *Aplysina*. J. Biol. Chem..

[78] Dogovic N., Sladic D., Kljiajic Z., Poznanovic S., Gasic M.J. (1996). Isolation and partial characterization of a lectin from the marine sponge *Crambe crambe*. J. Serb. Chem. Soc..

[79] Buck F., Luth C., Strupat K., Bretting H. (1992). Comparative investigations on the amino-acid sequences of different isolectins from the sponge *Axinella polypoides* (Schmidt). Biochim. Biophys. Acta.

[80] Hazes B. (1996). The (QxW)_3_ domain: A flexible lectin scaffold. Protein Sci..

[81] Kawagishi H., Yamawaki M., Isobe S., Usui T., Kimura A., Chiba S. (1994). Two lectins from the marine sponge *Halichondria okadai*. An *N*-acetyl-sugar-specific lectin (HOL-I) and an *N*-acetyllactosamine-specific lectin (HOL-II). J. Biol. Chem..

[82] Srivastava M., Simakov O., Chapman J., Fahey B., Gauthier M.E., Mitros T., Richards G.S., Conaco C., Dacre M., Hellsten U. (2010). The *Amphimedon queenslandica* genome and the evolution of animal complexity. Nature.

[83] Carneiro R.F., de Melo A.A., de Almeida A.S., Moura Rda M., Chaves R.P., de Sousa R.P., Nascimento K.S., Sampaio S.S., Lima J.P., Cavada B.S. (2013). H-3, a new lectin from the marine sponge *Haliclona caerulea*: Purification and mass spectrometric characterization. Int. J. Biochem. Cell Biol..

[84] Atta A.M., Barral-Netto M., Peixinho S., Sousa-Atta M.L. (1989). Isolation and functional characterization of a mitogenic lectin from the marine sponge *Cinachyrella alloclada*. Braz. J. Med. Biol. Res..

[85] Schröder H.C., Kljajic Z., Weiler B.E., Gasic M., Uhlenbruck G., Kurelec B., Müller W.E.G. (1990). The galactose-specific lectin from the sponge *Chondrilla nucula* displays anti-human immunodeficiency virus activity *in vitro* via stimulation of the (2′-5′) oligoadenylate metabolism. Antivir. Chem. Chemother..

[86] Atta A.M., Menezes E.P., Peixinho S., Sousa-Atta M.L. (1990). Isolation of a lectin from the marine sponge *Desmapsama anchorata* by affinity chromatography on raffinose-sepharose 6B. Braz. J. Med. Biol. Res..

[87] Bretting H., Jacobs G., Donadey C., Vacelet J. (1983). Immunohistochemical studies on the distribution and the function of the d-galactose-specific lectins in the sponge *Axinella polypoides* (Schmidt). Cell Tissue Res..

[88] Kaltner H., Stierstorfer B. (1998). Animal lectins as cell adhesion molecules. Acta Anat. Basel.

[89] Odintsova N.A., Belogortseva N.I., Ermak A.V., Molchanova V.I., Luk’yanov P.A. (1999). Adhesive and growth properties of lectin from the ascidian *Didemnum ternatanum* on cultivated marine invertebrate cells. Biochim. Biophys. Acta.

[90] Conrad J., Zahn R.K., Kurelec B., Uhlenbruck G., Müller W.E.G. (1981). Aggregation of sponge cells: Immunological characterization of the species-specific *Geodia* aggregation factor. J. Supramol. Struct. Cell. Biochem..

[91] Müller W.E.G., Conrad J., Zahn R.K., Gramzow M., Kurelec B., Uhlenbruck G. (1985). Identification and isolation of the primary aggregation factor from the cell membrane of the sponge *Geodia cydonium*. Mol. Cell. Biochem..

[92] Schütze J., Krasko A., Diehl-Seifert B., Müller W.E.G. (2001). Cloning and expression of the putative aggregation factor from the marine sponge *Geodia cydonium*. J. Cell. Sci..

[93] Blumbach B., Pancer Z., Diehl-Seifert B., Steffen R., Münkner J., Müller I.M., Müller W.E.G. (1998). The putative sponge aggregation receptor. Isolation and characterization of a molecule composed of scavenger receptor cysteine-rich domains and short consensus repeats. J. Cell Sci..

[94] Müller W.E.G., Conrad J., Zahn R.K., Steffen R., Uhlenbruck G., Müller I.M. (1984). Cell adhesion molecules in the hexactinellid *Aphrocallistes vastus*: Species-unspecific aggregation factor. Differentiation.

[95] McEver R.P. (2002). Selectins: Lectins that initiate cell adhesion under flow. Curr. Opin. Cell Biol..

[96] Phillips M.L., Nudelman E., Gaeta F.C., Perez M., Singhal A.K., Hakomori S., Paulson J.C. (1990). ELAM-1 mediates cell adhesion by recognition of a carbohydrate ligand, sialyl-Lex. Science.

[97] Fujii N., Minetti C.A., Nakhasi H.L., Chen S.W., Barbehenn E., Nunes P.H., Nguyen N.Y. (1992). Isolation, cDNA cloning, and characterization of an 18-kDa hemagglutinin and amebocyte aggregation factor from *Limulus polyphemus*. J. Biol. Chem..

[98] Yang J., Qiu L., Wei X., Wang L., Wang L., Zhou Z., Zhang H., Liu L., Song L. (2010). An ancient C-type lectin in *Chlamys farreri* (CfLec-2) that mediate pathogen recognition and cellular adhesion. Dev. Comp. Immunol..

[99] Ao J., Ling E., Yu X.Q. (2007). *Drosophila* C-type lectins enhance cellular encapsulation. Mol. Immunol..

[100] Wang L., Wang L., Kong P., Yang J., Zhang H., Wang M., Zhou Z., Qiu L., Song L. (2012). A novel C1qDC protein acting as pattern recognition receptor in scallop *Argopecten irradians*. Fish Shellfish Immunol..

[101] Wiens M., Schröder H.C., Wang X., Link T., Steindorf D., Müller W.E.G. (2011). Isolation of the silicatein-α interactor silintaphin-2 by a novel solid-phase pull-down assay. Biochemistry.

[102] Mann K., Siedler F. (2004). Ostrich (*Struthio camelus*) eggshell matrix contains two different C-type lectin-like proteins. Isolation, amino acid sequence, and posttranslational modifications. Biochim. Biophys. Acta.

[103] Lakshminarayanan R., Kini R.M., Valiyaveettil S. (2002). Investigation of the role of ansocalcin in the biomineralization in goose eggshell matrix. Proc. Natl. Acad. Sci. USA.

[104] Weiss I.M., Kaufmann S., Mann K., Fritz M. (2000). Purification and characterization of perlucin and perlustrin, two new proteins from the shell of the mollusc *Haliotis laevigata*. Biochem. Biophys. Res. Commun..

[105] Mann K., Weiss I.M., André S., Gabius H.J., Fritz M. (2000). The amino-acid sequence of the abalone (*Haliotis laevigata*) nacre protein perlucin. Detection of a functional C-type lectin domain with galactose/mannose specificity. Eur. J. Biochem..

[106] Naganuma T., Hoshino W., Shikanai Y., Sato R., Liu K., Sato S., Muramoto K., Osada M., Yoshimi K., Ogawa T. (2014). Novel matrix proteins of *Pteria penguin* pearl oyster shell nacre homologous to the jacalin-related β-prism fold lectins. PLoS ONE.

[107] Matsubara H., Hayashi T., Ogawa T., Muramoto K., Jimbo M., Kamiya H. (2008). Modulating effect of acorn barnacle C-type lectins on the crystallization of calcium carbonate. Fish Sci..

[108] Wiens M., Korzhev M., Krasko A., Thakur N.L., Perović-Ottstadt S., Breter H.J., Ushijima H., Diehl-Seifert B., Müller I.M., Müller W.E.G. (2005). Innate immune defense of the sponge *Suberites domuncula* against bacteria involves a MyD88-dependent signaling pathway. Induction of a perforin-like molecule. J. Biol. Chem..

[109] Wiens M., Korzhev M., Perovic-Ottstadt S., Luthringer B., Brandt D., Klein S., Müller W.E.G. (2007). Toll-like receptors are part of the innate immune defense system of sponges (demospongiae: Porifera). Mol. Biol. Evol..

[110] Thakur N., Perovic-Ottstadt S., Batel R., Korzhev M., Diehl-Seifert B., Müller I.M., Müller W.E.G. (2005). Innate immune defense of the sponge *Suberites domuncula* against gram-positive bacteria: Induction of lysozyme and AdaPTin. Mar. Biol..

[111] Schröder H.C., Natalio F., Wiens M., Tahir M.N., Shukoor M.I., Tremel W., Belikov S.I., Krasko A., Müller W.E.G. (2008). The 2′-5′-oligoadenylate synthetase in the lowest metazoa: Isolation, cloning, expression and functional activity in the sponge *Lubomirskia baicalensis*. Mol. Immunol..

[112] Perović-Ottstadt S., Adell T., Proksch P., Wiens M., Korzhev M., Gamulin V., Müller I.M., Müller W.E.G. (2008). A (1→3)-beta-d-glucan recognition protein from the sponge *Suberites domuncula*. Mediated activation of fibrinogen-like protein and epidermal growth factor gene expression. Mol. Immunol..

[113] Peumans W.J., van Damme E.J. (1995). Lectins as plant defense proteins. Plant Physiol..

[114] Charungchitrak S., Petsom A., Sangvanich P., Karnchanatat A. (2011). Antifungal and antibacterial activities of lectin from the seeds of *Archidendron jiringa* Nielsen. Food Chem..

[115] Lis H., Sharon N. (1986). Lectins as molecules and as tools. Nature.

[116] Vandenborre G., Smagghe G., van Damme E.J. (2011). Plant lectins as defense proteins against phytophagous insects. Phytochemistry.

[117] Gaynor C.D., McCormack F.X., Voelker D.R., McGowan S.E., Schlesinger L.S. (1995). Pulmonary surfactant protein A mediates enhanced phagocytosis of *Mycobacterium tuberculosis* by a direct interaction with human macrophages. J. Immunol..

[118] McNeely T.B., Coonrod J.D. (1993). Comparison of the opsonic activity of human surfactant protein A for *Staphylococcus aureus* and *Streptococcus pneumoniae* with rabbit and human macrophages. J. Infect. Dis..

[119] Matsushita M., Endo Y., Taira S., Sato Y., Fujita T., Ichikawa N., Nakata M., Mizuochi T. (1996). A novel human serum lectin with collagen- and fibrinogen-like domains that functions as an opsonin. J. Biol. Chem..

[120] Wang L., Wang L., Zhang D., Jiang Q., Sun R., Wang H., Zhang H., Song L. (2015). A novel multi-domain C1qDC protein from Zhikong scallop *Chlamys farreri* provides new insights into the function of invertebrate C1qDC proteins. Dev. Comp. Immunol..

[121] Jiang S., Li H., Zhang D., Zhang H., Wang L., Sun J., Song L. (2015). A C1q domain containing protein from *Crassostrea gigas* serves as pattern recognition receptor and opsonin with high binding affinity to LPS. Fish Shellfish Immunol..

[122] Takahashi K.G., Kuroda T., Muroga K. (2008). Purification and antibacterial characterization of a novel isoform of the Manila clam lectin (MCL-4) from the plasma of the Manila clam, *Ruditapes philippinarum*. Comp. Biochem. Physiol. B Biochem. Mol. Biol..

[123] Sun Y.D., Fu L.D., Jia Y.P., Du X.J., Wang Q., Wang Y.H., Zhao X.F., Yu X.Q., Wang J.X. (2008). A hepatopancreas-specific C-type lectin from the Chinese shrimp *Fenneropenaeus chinensis* exhibits antimicrobial activity. Mol. Immunol..

[124] Hentschel U., Usher K.M., Taylor M.W. (2006). Marine sponges as microbial fermenters. FEMS Microbiol. Ecol..

[125] Wehrl M., Steinert M., Hentschel U. (2007). Bacterial uptake by the marine sponge *Aplysina aerophoba*. Microb. Ecol..

[126] Reveillaud J., Maignien L., Eren A.M., Huber J.A., Apprill A., Sogin M.L., Vanreusel A. (2014). Host-specificity among abundant and rare taxa in the sponge microbiome. ISME J..

[127] Fieseler L., Horn M., Wagner M., Hentschell U. (2004). Discovery of the novel candidate phylum “Poribacteria” in marine sponges. Appl. Environ. Microbiol..

[128] Siegl A., Kamke J., Hochmuth T., Piel J., Richter M., Liang C., Dandekar T., Hentschel U. (2011). Single-cell genomics reveals the lifestyle of Poribacteria, a candidate phylum symbiotically associated with marine sponges. ISME J..

[129] Thomas T., Rusch D., Demaere M.Z., Yung P.Y., Lewis M., Halpern A., Heidelberg K.B., Egan S., Steinberg P.D., Kjelleberg S. (2010). Functional genomic signatures of sponge bacteria reveal unique and shared features of symbiosis. ISME J..

[130] Gardères J., Henry J., Bernay B., Ritter A., Zatylny-Gaudin C., Wiens M., Müller W.E.G., Le Pennec G. (2014). Cellular effects of bacterial *N*-3-oxo-dodecanoyl-l-homoserine lactone on the sponge *Suberites domuncula* (Olivi, 1792): Insights into an intimate inter-kingdom dialogue. PLoS ONE.

[131] Bry L., Falk P.G., Midtvedt T., Gordon J.I. (1996). A model of host-microbial interactions in an open mammalian ecosystem. Science.

[132] Hooper L.V., Falk P.G., Gordon J.I. (2000). Analyzing the molecular foundations of commensalism in the mouse intestine. Curr. Opin. Microbiol..

[133] Martens E.C., Goodrich-Blair H. (2005). The *Steinernema carpocapsae* intestinal vesicle contains a subcellular structure with which *Xenorhabdus nematophila* associates during colonization initiation. Cell Microbiol..

[134] Nyholm S.V., Stabb E.V., Ruby E.G., McFall-Ngai M.J. (2000). Establishment of an animal-bacterial association: Recruiting symbiotic *vibrios* from the environment. Proc. Natl. Acad. Sci. USA.

[135] Vydryakova G.A., Bondar’ V.S. (2008). Location of lectin exhibiting specificity for *N*-acetyl-d-galactosamine in cells of the symbiotic marine bacteria *Photobacterium phosphoreum*. Dokl. Biochem. Biophys..

[136] Jimbo M., Yanohara T., Koike K., Sakai R., Muramoto K., Kamiya H. (2000). The d-galactose-binding lectin of the octocoral *Sinularia lochmodes*: Characterization and possible relationship to the symbiotic dinoflagellates. Comp. Biochem. Physiol. B Biochem. Mol. Biol..

[137] Bulgheresi S., Schabussova I., Chen T., Mullin N.P., Maizels R.M., Ott J.A. (2006). A new C-type lectin similar to the human immunoreceptor DC-SIGN mediates symbiont acquisition by a marine nematode. Appl. Environ. Microbiol..

[138] Zhang P., Snyder S., Feng P., Azadi P., Zhang S., Bulgheresi S., Sanderson K.E., He J., Klena J., Chen T. (2006). Role of *N*-acetylglucosamine within core lipopolysaccharide of several species of gram-negative bacteria in targeting the DC-SIGN (CD209). J. Immunol..

[139] De Hoff P.L., Brill L.M., Hirsch A.M. (2009). Plant lectins: The ties that bind in root symbiosis and plant defense. Mol. Genet. Genom..

[140] Van Rhijn P., Fujishige N.A., Lim P.O., Hirsch A.M. (2001). Sugar-binding activity of pea lectin enhances heterologous infection of transgenic alfalfa plants by *Rhizobium leguminosarum* biovar viciae. Plant Physiol..

[141] Laus M.C., Logman T.J., Lamers G.E., van Brussel A.A., Carlson R.W., Kijne J.W. (2006). A novel polar surface polysaccharide from *Rhizobium leguminosarum* binds host plant lectin. Mol. Microbiol..

[142] Queiroz A.F., Moura R.M., Ribeiro J.K., Lyra I.L., Cunha D.C., Santos E.A., de-Sales M.P. (2008). Pro-inflammatory effect in mice of CvL, a lectin from the marine sponge *Cliona varians*. Comp. Biochem. Physiol. C Toxicol. Pharmacol..

[143] Yamaoka A., Kuwabara I., Frigeri L.G., Liu F.T. (1995). A human lectin, galectin-3 (epsilon bp/Mac-2), stimulates superoxide production by neutrophils. J. Immunol..

[144] Hsu D.K., Yang R.Y., Pan Z., Yu L., Salomon D.R., Fung-Leung W.P., Liu F.T. (2000). Targeted disruption of the galectin-3 gene results in attenuated peritoneal inflammatory responses. Am. J. Pathol..

[145] Sano H., Hsu D.K., Yu L., Apgar J.R., Kuwabara I., Yamanaka T., Hirashima M., Liu F.T. (2000). Human galectin-3 is a novel chemoattractant for monocytes and macrophages. J. Immunol..

[146] Okino N., Kawabata S., Saito T., Hirata M., Takagi T., Iwanaga S. (1995). Purification, characterization, and cDNA cloning of a 27-kDa lectin (L10) from horseshoe crab hemocytes. J. Biol. Chem..

[147] Kawabata S., Iwanaga S. (1999). Role of lectins in the innate immunity of horseshoe crab. Dev. Comp. Immunol..

[148] Saito T., Hatada M., Iwanaga S., Kawabata S. (1997). A newly identified horseshoe crab lectin with binding specificity to *O*-antigen of bacterial lipopolysaccharides. J. Biol. Chem..

[149] Inamori K., Saito T., Iwaki D., Nagira T., Iwanaga S., Arisaka F., Kawabata S. (1999). A newly identified horseshoe crab lectin with specificity for blood group A antigen recognizes specific *O*-antigens of bacterial lipopolysaccharides. J. Biol. Chem..

[150] Dey B., Lerner D.L., Lusso P., Boyd M.R., Elder J.H., Berger E.A. (2000). Multiple antiviral activities of cyanovirin-N: Blocking of human immunodeficiency virus type 1 gp120 interaction with CD4 and coreceptor and inhibition of diverse enveloped viruses. J. Virol..

[151] Jacobson R.L., Schlein Y. (1999). Lectins and toxins in the plant diet of *Phlebotomus papatasi* (Diptera: Psychodidae) can kill *Leishmania major* promastigotes in the sandfly and in culture. Ann. Trop. Med. Parasitol..

[152] Gazzinelli R.T., Pereira M.E., Romanha A., Gazzinelli G., Brener Z. (1991). Direct lysis of *Trypanosoma cruzi*: A novel effector mechanism of protection mediated by human anti-gal antibodies. Parasite Immunol..

[153] Jacobson R.L., Doyle R.J., Slifkin M. (1994). Lectin-*Leishmania* interaction. Lectin-Microorganisms Interaction.

[154] Schottelius J., Alsien M.S.O., Doyle R.J., Slifkin M. (1994). Lectin-Microorganisms Interaction.

[155] Castanheira L.E., Nunes D.C., Cardoso T.M., Santos Pde S., Goulart L.R., Rodrigues R.S., Richardson M., Borges M.H., Yoneyama K.A., Rodrigues V.M. (2013). Biochemical and functional characterization of a C-type lectin (BpLec) from *Bothrops pauloensis* snake venom. Int. J. Biol. Macromol..

[156] Afonso-Cardoso S.R., Rodrigues F.H., Gomes M.A., Silva A.G., Rocha A., Guimaraes A.H., Candeloro I., Favoreto S., Ferreira M.S., de Souza M.A. (2007). Protective effect of lectin from *Synadenium carinatum* on *Leishmania amazonensis* infection in BALB/c mice. Korean J. Parasitol..

[157] Queiroz A.F., Silva R.A., Moura R.M., Dreyfuss J.L., Paredes-Gamero E.J., Souza A.C., Tersariol I.L., Santos E.A., Nader H.B., Justo G.Z. (2009). Growth inhibitory activity of a novel lectin from *Cliona varians* against K562 human erythroleukemia cells. Cancer Chemother. Pharmacol..

[158] Rabelo L., Monteiro N., Serquiz R., Santos P., Oliveira R., Oliveira A., Rocha H., Morais A.H., Uchoa A., Santos E. (2012). A lactose-binding lectin from the marine sponge *Cinachyrella apion* (Cal) induces cell death in human cervical adenocarcinoma cells. Mar. Drugs.

[159] Bantel H., Engels I.H., Voelter W., Schulze-Osthoff K., Wesselborg S. (1999). Mistletoe lectin activates caspase-8/FLICE independently of death receptor signaling and enhances anticancer drug-induced apoptosis. Cancer Res..

[160] Opric M.M., Poznanovic S., Kljajic Z., Sladic D., Pupic G., Perunovic B., Gasic M.J. (1996). Labelling of breast carcinoma, thyroid carcinoma and melanoma with manno- and galacto-specific lectins from marine invertebrates. Eur. J. Histochem..

[161] Gorelik E., Galili U., Raz A. (2001). On the role of cell surface carbohydrates and their binding proteins (lectins) in tumor metastasis. Cancer Metastasis Rev..

[162] Müller W.E.G. (2001). Review: How was metazoan threshold crossed? The hypothetical Urmetazoa. A. Mol. Integr. Physiol..

[163] Barauna S.C., Kaster M.P., Heckert B.T., do Nascimento K.S., Rossi F.M., Teixeira E.H., Cavada B.S., Rodrigues A.L., Leal R.B. (2006). Antidepressant-like effect of lectin from *Canavalia brasiliensis* (ConBr) administered centrally in mice. Pharmacol. Biochem. Behav..

[164] Soares G.D., Lima C.B., Cavalcanti L.C., Villacampa N., Castellano B., Guedes R.C. (2015). Brain effects of the lectin from *Canavalia ensiformis* in adult rats previously suckled in favorable and unfavorable conditions: A spreading depression and microglia immunolabeling study. Nutr. Neurosci..

